# Supplementary material for: Eurasian back-migration into Northeast Africa was a complex and multifaceted process
Source: PLoS One. 2023 Nov 8;18(11):e0290423. doi: 10.1371/journal.pone.0290423 (PMC10631636; doi:10.1371/journal.pone.0290423)
Supplement: S1 Table — (PDF) [file pone.0290423.s001.pdf]

S Table 1: **Top two Eurasian source populations identified by their haplotype fit to the genomes (R^2 value from MOSAIC) for each target population.**

Target	Sources	R2
Ethiopia_AFAR	GIH-Gujarati_India & Lebanese_Christian	0.7097513
Ethiopia_AMHARA	Dubai_Dubai & GIH-Gujarati_India	0.7170762
Ethiopia_TYGRAY	GIH-Gujarati_India & Lebanese_Muslim	0.7086051
Ethiopia_WOLAYTA	FIN-Finish_Finland & GIH-Gujarati_India	0.7322527
Ethiopia_Oromo	FIN-Finish_Finland & Lebanese_Christian	0.7271779
Egypt_Egyptian	IBS-Iberian_Spain & Lebanese_Druze	0.8125434
Ethiopia_GUMUZ	Dubai_Dubai & Oman_Oman	0.765788
Ethiopia_ANUAK	Oman_Oman & SaudiArabia_SaudiArabia	0.7631948
Ethiopia_Somali	Dubai_Dubai & Iran_Iran	0.6993737
Somalia_Somali	GIH-Gujarati_India & Yemen_YEMEN	0.7882802
Kenya_Samburu	Dubai_Dubai & Oman_Oman	0.7269168
Kenya_Turkana	Dubai_Dubai & Yemen_YEMEN	0.8463287
Kenya_Kikuyu	Lebanese_Muslim & Yemen_YEMEN	0.7181286
MKK-Maasai_Kenya	SaudiArabia_SaudiArabia & TSI-Toscani_Itali	0.7204982
LWK-Luhya_Kenya	Oman_Oman & Qatar_Qatar	0.75834
Uganda_Baganda	Iran_Iran & Oman_Oman	0.7625496
Uganda_Banyarwanda	Lebanese_Muslim & Yemen_YEMEN	0.7379781
Uganda_Barundi	Lebanese_Muslim & TSI-Toscani_Itali	0.787222
Sudan_Baria	Dubai_Dubai & GIH-Gujarati_India	0.7562114
Sudan_Bataheen	GIH-Gujarati_India & Lebanese_Christian	0.8134197
Sudan_BeniAmer	GIH-Gujarati_India & Lebanese_Muslim	0.6988863
Sudan_Copt	IBS-Iberian_Spain & Lebanese_Christian	0.697059
Sudan_Danagla	IBS-Iberian_Spain & TSI-Toscani_Itali	0.7615013
Sudan_Gaalien	GBR-British_UK & Lebanese_Christian	0.7974334
Sudan_Gemar	Lebanese_Christian & Lebanese_Muslim	0.8498585
Sudan_Hadendowa	GIH-Gujarati_India & Iran_Iran	0.8009134
Sudan_Halfawieen	GIH-Gujarati_India & Lebanese_Christian	0.7735757
Sudan_Hausa	Dubai_Dubai & Oman_Oman	0.7649491
Sudan_Mahas	IBS-Iberian_Spain & TSI-Toscani_Itali	0.7600216
Sudan_Messiria	Iran_Iran & Lebanese_Druze	0.8687931
Sudan_Nuba	FIN-Finish_Finland & Lebanese_Druze	0.8584353
Sudan_Nuer	Oman_Oman & Yemen_YEMEN	0.7484863
Sudan_Shaigia	GIH-Gujarati_India & Yemen_YEMEN	0.8093127
Sudan_Shilluk	GIH-Gujarati_India & TSI-Toscani_Itali	0.758663
Sudan_Zagawa	Dubai_Dubai & Oman_Oman	0.7174841
