# Supplementary material for: Eurasian back-migration into Northeast Africa was a complex and multifaceted process
Source: PLoS One. 2023 Nov 8;18(11):e0290423. doi: 10.1371/journal.pone.0290423 (PMC10631636; doi:10.1371/journal.pone.0290423)
Supplement: S2 Table — The f3 outgroup was calculated for the most Eurasian-like ancestry for each target population in the following manner: Target | Source | Ju|’hoansi. (PDF) [file pone.0290423.s002.pdf]

S Table 2: F_3 outgroup result grouped by language family of the target populations, top 5 hits show. The F_3 outgroup was calculated for the most Eurasian-like ancestry for each target population in the following manner: Target | Source | Ju|'hoansi

Omotic		
Target Population	Source	F3-Value
Ethiopia_WOLAYTA	Lebanese_Christian	0,001883
Ethiopia_WOLAYTA	Lebanese_Druze	0,001899
Ethiopia_WOLAYTA	Yemen_YEMEN	0,001872
Ethiopia_WOLAYTA	TSI-Toscani_Italy	0,001892
Ethiopia_WOLAYTA	IBS-Iberian_Spain	0,00191

Cushitic		
Target Population	Source	F3-Value
Ethiopia_AFAR	Lebanese_Christian	0,001832
Ethiopia_AFAR	Yemen_YEMEN	0,001856
Ethiopia_AFAR	Lebanese_Druze	0,001836
Ethiopia_AFAR	TSI-Toscani_Italy	0,001811
Ethiopia_AFAR	IBS-Iberian_Spain	0,001818
Ethiopia_Oromo	Lebanese_Christian	0,001783
Ethiopia_Oromo	Lebanese_Druze	0,00178
Ethiopia_Oromo	Yemen_YEMEN	0,001801
Ethiopia_Oromo	TSI-Toscani_Italy	0,001763
Ethiopia_Oromo	IBS-Iberian_Spain	0,001764
Ethiopia_Somali	Lebanese_Christian	0,001937
Ethiopia_Somali	Yemen_YEMEN	0,001958
Ethiopia_Somali	Lebanese_Druze	0,001967
Ethiopia_Somali	TSI-Toscani_Italy	0,001957
Ethiopia_Somali	IBS-Iberian_Spain	0,00195
Somalia_Somali	Lebanese_Christian	0,001919
Somalia_Somali	Yemen_YEMEN	0,001906
Somalia_Somali	Lebanese_Druze	0,0019
Somalia_Somali	TSI-Toscani_Italy	0,001935
Somalia_Somali	IBS-Iberian_Spain	0,001928

Languageisolate		
Target Population	Source	F3-Value
Ethiopia_GUMUZ	Lebanese_Christian	0,00363
Ethiopia_GUMUZ	FIN-Finish_Finland	0,00353
Ethiopia_GUMUZ	Lebanese_Druze	0,003529
Ethiopia_GUMUZ	TSI-Toscani_Italy	0,003478
Ethiopia_GUMUZ	IBS-Iberian_Spain	0,003447

Chadica		
Target Population	Source	F3-Value
Sudan_Hausa	TSI-Toscani_Italy	0,00374
Sudan_Hausa	IBS-Iberian_Spain	0,003698
Sudan_Hausa	Lebanese_Christian	0,003736
Sudan_Hausa	Lebanese_Druze	0,003774
Sudan_Hausa	GBR-British_UK	0,003634

Saharan		
Target Population	Source	F3-Value
Sudan_Gemar	Lebanese_Christian	0,002454
Sudan_Gemar	Lebanese_Druze	0,002469
Sudan_Gemar	TSI-Toscani_Italy	0,002464
Sudan_Gemar	Yemen_YEMEN	0,002435
Sudan_Gemar	IBS-Iberian_Spain	0,002454
Sudan_Zagawa	TSI-Toscani_Italy	0,002557
Sudan_Zagawa	IBS-Iberian_Spain	0,002564
Sudan_Zagawa	GBR-British_UK	0,002553
Sudan_Zagawa	Lebanese_Christian	0,00256
Sudan_Zagawa	Lebanese_Druze	0,002543

Niger-Congo/Bantoid		
Target Population	Source	F3-Value
Kenya_Kikuyu	Lebanese_Christian	0,002319
Kenya_Kikuyu	TSI-Toscani_Italy	0,002327
Kenya_Kikuyu	Lebanese_Druze	0,002314
Kenya_Kikuyu	Lebanese_Muslim	0,002282
Kenya_Kikuyu	Yemen_YEMEN	0,002345
LWK-Luhya_Kenya	Lebanese_Muslim	0,003496
LWK-Luhya_Kenya	IBS-Iberian_Spain	0,003503
LWK-Luhya_Kenya	TSI-Toscani_Italy	0,003523
LWK-Luhya_Kenya	Lebanese_Christian	0,003566
LWK-Luhya_Kenya	Lebanese_Druze	0,003512
Uganda_Baganda	IBS-Iberian_Spain	0,00442
Uganda_Baganda	Yemen_YEMEN	0,004379
Uganda_Baganda	TSI-Toscani_Italy	0,004346
Uganda_Baganda	Lebanese_Christian	0,004306
Uganda_Baganda	Lebanese_Druze	0,004285
Uganda_Banyarwanda	Lebanese_Christian	0,002659
Uganda_Banyarwanda	TSI-Toscani_Italy	0,002623
Uganda_Banyarwanda	IBS-Iberian_Spain	0,002637
Uganda_Banyarwanda	Lebanese_Druze	0,002626
Uganda_Banyarwanda	Yemen_YEMEN	0,002646
Uganda_Barundi	Lebanese_Christian	0,002701
Uganda_Barundi	TSI-Toscani_Italy	0,002675
Uganda_Barundi	IBS-Iberian_Spain	0,002684
Uganda_Barundi	Lebanese_Druze	0,002688
Uganda_Barundi	Yemen_YEMEN	0,002674

Nilotic		
Target Population	Source	F3-Value
Kenya_Samburu	Lebanese_Christian	0,001817
Kenya_Samburu	Yemen_YEMEN	0,001804
Kenya_Samburu	Lebanese_Druze	0,001851
Kenya_Samburu	TSI-Toscani_Italy	0,001831
Kenya_Samburu	IBS-Iberian_Spain	0,001832
Kenya_Turkana	Lebanese_Christian	0,002338
Kenya_Turkana	Lebanese_Druze	0,002354
Kenya_Turkana	Yemen_YEMEN	0,002334
Kenya_Turkana	TSI-Toscani_Italy	0,002338
Kenya_Turkana	Lebanese_Muslim	0,002321
MKK-Maasai_Kenya	TSI-Toscani_Italy	0,001968
MKK-Maasai_Kenya	Lebanese_Christian	0,001974
MKK-Maasai_Kenya	Lebanese_Druze	0,001952
MKK-Maasai_Kenya	IBS-Iberian_Spain	0,001956
MKK-Maasai_Kenya	Yemen_YEMEN	0,001947
Ethiopia_ANUAK	Lebanese_Christian	0,005159
Ethiopia_ANUAK	Lebanese_Druze	0,005166
Ethiopia_ANUAK	IBS-Iberian_Spain	0,005093
[illegible]	[illegible]	[illegible]

Semitic		
Target Population	Source	F3-Value
Egypt_Egyptian	Lebanese_Christian	0,001853
Egypt_Egyptian	Lebanese_Druze	0,001865
Egypt_Egyptian	TSI-Toscani_Italy	0,001852
Egypt_Egyptian	IBS-Iberian_Spain	0,001849
Egypt_Egyptian	GBR-British_UK	0,001841
Ethiopia_AMHARA	Lebanese_Christian	0,001742
Ethiopia_AMHARA	Yemen_YEMEN	0,001748
Ethiopia_AMHARA	Lebanese_Druze	0,001753
Ethiopia_AMHARA	Lebanese_Muslim	0,001723
Ethiopia_AMHARA	TSI-Toscani_Italy	0,001741
Ethiopia_TYGRAY	Lebanese_Christian	0,001768
Ethiopia_TYGRAY	Lebanese_Druze	0,001757
Ethiopia_TYGRAY	Yemen_YEMEN	0,001787
Ethiopia_TYGRAY	TSI-Toscani_Italy	0,001759
Ethiopia_TYGRAY	Lebanese_Muslim	0,00175
Sudan_Bataheen	Lebanese_Christian	0,001855
Sudan_Bataheen	Lebanese_Druze	0,001859
Sudan_Bataheen	TSI-Toscani_Italy	0,001837
Sudan_Bataheen	IBS-Iberian_Spain	0,001837
Sudan_Bataheen	Yemen_YEMEN	0,001851
Sudan_BeniAmer	Lebanese_Christian	0,001871
Sudan_BeniAmer	Lebanese_Druze	0,001857
Sudan_BeniAmer	Yemen_YEMEN	0,001873
Sudan_BeniAmer	TSI-Toscani_Italy	0,001862
Sudan_BeniAmer	IBS-Iberian_Spain	0,001858
Sudan_Gaalien	Lebanese_Christian	0,001844
Sudan_Gaalien	Lebanese_Druze	0,001851
Sudan_Gaalien	TSI-Toscani_Italy	0,001863
Sudan_Gaalien	Yemen_YEMEN	0,001868
Sudan_Gaalien	IBS-Iberian_Spain	0,001861
Sudan_Hadendowa	Lebanese_Christian	0,001838
Sudan_Hadendowa	Lebanese_Druze	0,001837
Sudan_Hadendowa	Sudan_Hadendowa	0,001776
Sudan_Hadendowa	Yemen_YEMEN	0,001827
Sudan_Hadendowa	TSI-Toscani_Italy	0,001828
Sudan_Messiria	TSI-Toscani_Italy	0,002089
Sudan_Messiria	Lebanese_Christian	0,002128
Sudan_Messiria	Lebanese_Druze	0,002077
Sudan_Messiria	IBS-Iberian_Spain	0,002093
Sudan_Messiria	Lebanese_Muslim	0,002078
Sudan_Shaigia	Lebanese_Christian	0,001851
Sudan_Shaigia	Lebanese_Druze	0,001855
Sudan_Shaigia	TSI-Toscani_Italy	0,001834
Sudan_Shaigia	Yemen_YEMEN	0,001861
Sudan_Shaigia	IBS-Iberian_Spain	0,001831

Egyptian		
Target Population	Source	F3-Value
Sudan_Copt	Lebanese_Christian	0,001847
Sudan_Copt	Lebanese_Druze	0,001851
Sudan_Copt	TSI-Toscani_Italy	0,001839
Sudan_Copt	IBS-Iberian_Spain	0,001839
Sudan_Copt	GBR-British_UK	0,001826

EasternSudanic		
Target Population	Source	F3-Value
Sudan_Danagla	Lebanese_Christian	0,001827
Sudan_Danagla	TSI-Toscani_Italy	0,00183
Sudan_Danagla	Lebanese_Druze	0,001806
Sudan_Danagla	IBS-Iberian_Spain	0,001809
Sudan_Danagla	GBR-British_UK	0,001799
Sudan_Halfawieen	Lebanese_Christian	0,00189
Sudan_Halfawieen	TSI-Toscani_Italy	0,001895
Sudan_Halfawieen	Lebanese_Druze	0,001883
Sudan_Halfawieen	IBS-Iberian_Spain	0,001884
Sudan_Halfawieen	GBR-British_UK	0,001878
Sudan_Mahas	Lebanese_Christian	0,001848
Sudan_Mahas	Lebanese_Druze	0,001843
Sudan_Mahas	TSI-Toscani_Italy	0,001818
Sudan_Mahas	IBS-Iberian_Spain	0,001834
Sudan_Mahas	Lebanese_Muslim	0,001822
Sudan_Nuba	Lebanese_Christian	0,002383
Sudan_Nuba	TSI-Toscani_Italy	0,002394
Sudan_Nuba	Lebanese_Druze	0,002367
Sudan_Nuba	IBS-Iberian_Spain	0,002403
Sudan_Nuba	Lebanese_Muslim	0,002352
Sudan_Shilluk	TSI-Toscani_Italy	0,004437
Sudan_Shilluk	Lebanese_Christian	0,004543
Sudan_Shilluk	Lebanese_Druze	0,004445
Sudan_Shilluk	IBS-Iberian_Spain	0,0045
Sudan_Shilluk	SaudiArabia_SaudiArabia	0,004459
Sudan_Baria	Qatar_Qatar	0,006987
Sudan_Baria	Lebanese_Muslim	0,007209
Sudan_Baria	GBR-British_UK	0,007117
Sudan_Baria	IBS-Iberian_Spain	0,00734
Sudan_Baria	TSI-Toscani_Italy	0,007282
Sudan_Nuer	GBR-British_UK	0,003912
Sudan_Nuer	FIN-Finish_Finland	0,00397
Sudan_Nuer	IBS-Iberian_Spain	0,003864
Sudan_Nuer	TSI-Toscani_Italy	0,003808
Sudan_Nuer	Lebanese_Christian	0,003829
