# Supplementary material for: Eurasian back-migration into Northeast Africa was a complex and multifaceted process
Source: PLoS One. 2023 Nov 8;18(11):e0290423. doi: 10.1371/journal.pone.0290423 (PMC10631636; doi:10.1371/journal.pone.0290423)
Supplement: S3 Table — The Best by f3 dataset is in the leftmost columns while the Best by R2 dataset is on the right. Some linguistic groups have only one target population so no value whilst Saharan two populations which yields a R2 of 1. (PDF) [file pone.0290423.s003.pdf]

S Table 3: R^2 values fof the linear regression between admixture date and distance from Tel Aviv in the top row by each linguistic group. The Best by F_3 dataset is in the leftmost columns while the Best by R^2 dataset is on the right. Some linguistic groups have only one target population so no value while Saharan two populations which yields a R^2 of 1.

Distance from Tel Aviv			
Best by F3		Best by F2	
Linguistic group	R2	Linguistic group	R2
African_semitic	0.002937986	African_semitic	0.010044898
Cushitic	0.303608661	Cushitic	0.383364613
Nilotic	0.138060608	Nilotic	0.967341737
Languageisolate	-	Languageisolate	-
Omotic	-	Omotic	-
Bantoid	0.003136976	Bantoid	0.006483384
EasternSudanic	0.830858222	Egyptian	-
Egyptian	-	EasternSudanic	0.033576907
Saharan	1.000000000	Saharan	1.000000000
Chadica	-	Chadica	-
Distance from Sanaa			
Best by F3		Best by R2	
Linguistic group	R2	Linguistic group	R2
African_semitic	0.02863027	African_semitic	0.04740316
Cushitic	0.26499817	Cushitic	0.43091650
Nilotic	0.16364704	Nilotic	0.98158939
Languageisolate	-	Languageisolate	0.00000000
Omotic	-	Omotic	0.00000000
Bantoid	0.06104717	Bantoid	0.42390308
EasternSudanic	0.82342297	Egyptian	0.00000000
Egyptian	-	EasternSudanic	0.07372633
Saharan	1.00000000	Saharan	1.00000000
Chadica	-	Chadica	-
