# Supplementary material for: Eurasian back-migration into Northeast Africa was a complex and multifaceted process
Source: PLoS One. 2023 Nov 8;18(11):e0290423. doi: 10.1371/journal.pone.0290423 (PMC10631636; doi:10.1371/journal.pone.0290423)
Supplement: S4 Table — Comparisons are between the Admixture date with the factors Country, Linguistic group and Larger linguistic family (Meta.Lang.Group). The Asterisk indicates significant values. In A it’s the admixture date obtained from the best source determined by f3 outgroup and in B it’s the dates of admixture for the source with the highest. (PDF) [file pone.0290423.s004.pdf]

S Table 4: Two way ANOVA statistics for the two different models, comparing the Admixture date with the factors Country, Linguistic group and Larger linguistic family (Meta.Lang.Group). The Asterisk indicates significant values. In A it's the admixture date obtained from the best source determined by F_3 outgroup and in B it's the dates of admixture for the source with the highest R^2 value.

A	By F3				
	Df	Sum_Sq	Mean_Sq	F_value	Pr(>F)
factor(Meta.Lang.Group)	3	696.9	232.29	3.514	0.0352*
factor(Lang.Group)	6	177.1	29.51	0.446	0.8385
factor(Country)	6	530.7	88.45	1.338	0.2890
Residuals	19	1255.9	66.10		
B	By R2				
	Df	Sum_Sq	Mean_Sq	F_value	Pr(>F)
factor(Meta.Lang.Group)	3	493.3	164.42	3.038	0.0595
factor(Lang.Group)	5	433.0	86.59	1.600	0.2166
factor(Country)	5	211.3	42.26	0.781	0.5780
Residuals	16	866.0	54.12		
