# Supplementary material for: Eurasian back-migration into Northeast Africa was a complex and multifaceted process
Source: PLoS One. 2023 Nov 8;18(11):e0290423. doi: 10.1371/journal.pone.0290423 (PMC10631636; doi:10.1371/journal.pone.0290423)
Supplement: S5 Table — Min and max is the individual lowest and highest missingness for that population. Since non-Eurasian regions of the target’s genomes were set to missing, this measure is the inverse of the amount of Eurasian ancestry inferred for each individual and population in the best by f3 dataset. (PDF) [file pone.0290423.s005.pdf]

S Table 5: **Missingness by population for the Ancestry deconvolution** Min and max is the individual lowest and highest missingness for that population. Since non-Eurasian regions of the target's genomes were set to missing, this measure is the inverse of the amount of Eurasian ancestry inferred for each individual and population in the best by f_3 dataset.

Target	Mean	Min	Max
Ethiopia_Afar	0.687	0.549	0.762
Ethiopia_Amhara	0.666	0.582	0.77
Ethiopia_Somali	0.666	0.600	0.748
Egypt_Egyptian	0.287	0.236	0.357
Ethiopia_Oromo	0.643	0.518	0.765
Ethiopia_Somali	0.683	0.581	0.793
Ethiopia_Gumuz	0.984	0.932	0.999
Kenya_Kikuyu	0.822	0.720	0.882
Kenya_Samburu	0.704	0.611	0.775
Kenya_Turkana	0.807	0.475	0.945
LWK	0.967	0.940	0.986
MKK	0.816	0.766	0.878
Ethiopia_Oromo	0.641	0.489	0.827
Somalia_Somali	0.705	0.535	0.926
Sudan_Bataheen	0.544	0.428	0.774
Sudan_BeniAmer	0.903	0.789	1
Sudan_Copt	1.00	0.999	1
Sudan_Danagla	0.581	0.464	0.672
Sudan_Gaalien	0.614	0.492	0.680
Sudan_Gemar	0.866	0.616	0.990
Sudan_Hadendowa	0.614	0.460	0.978
Sudan_Halfawieen	0.507	0.378	0.602
Sudan_Hausa	0.961	0.937	0.986
Sudan_Mahas	0.548	0.474	0.617
Sudan_Messiria	0.813	0.503	0.977
Sudan_Nuba	0.935	0.448	0.997
Sudan_Shaigia	0.588	0.463	0.926
Sudan_Shilluk	0.987	0.950	1.00
Sudan_Zagawa	0.930	0.845	0.985
Ethiopia_Tygray	0.656	0.576	0.771
Uganda_Baganda	0.982	0.965	0.998
Uganda_Banyarwanda	0.913	0.822	0.959
Uganda_Barundi	0.918	0.613	0.987
Ethiopia_Wolayta	0.711	0.611	0.845
