# Supplementary material for: Eurasian back-migration into Northeast Africa was a complex and multifaceted process
Source: PLoS One. 2023 Nov 8;18(11):e0290423. doi: 10.1371/journal.pone.0290423 (PMC10631636; doi:10.1371/journal.pone.0290423)
Supplement: S1 Fig — Colours indicate linguistic groups. Made with Natural Earth. (PDF) [file pone.0290423.s007.pdf]

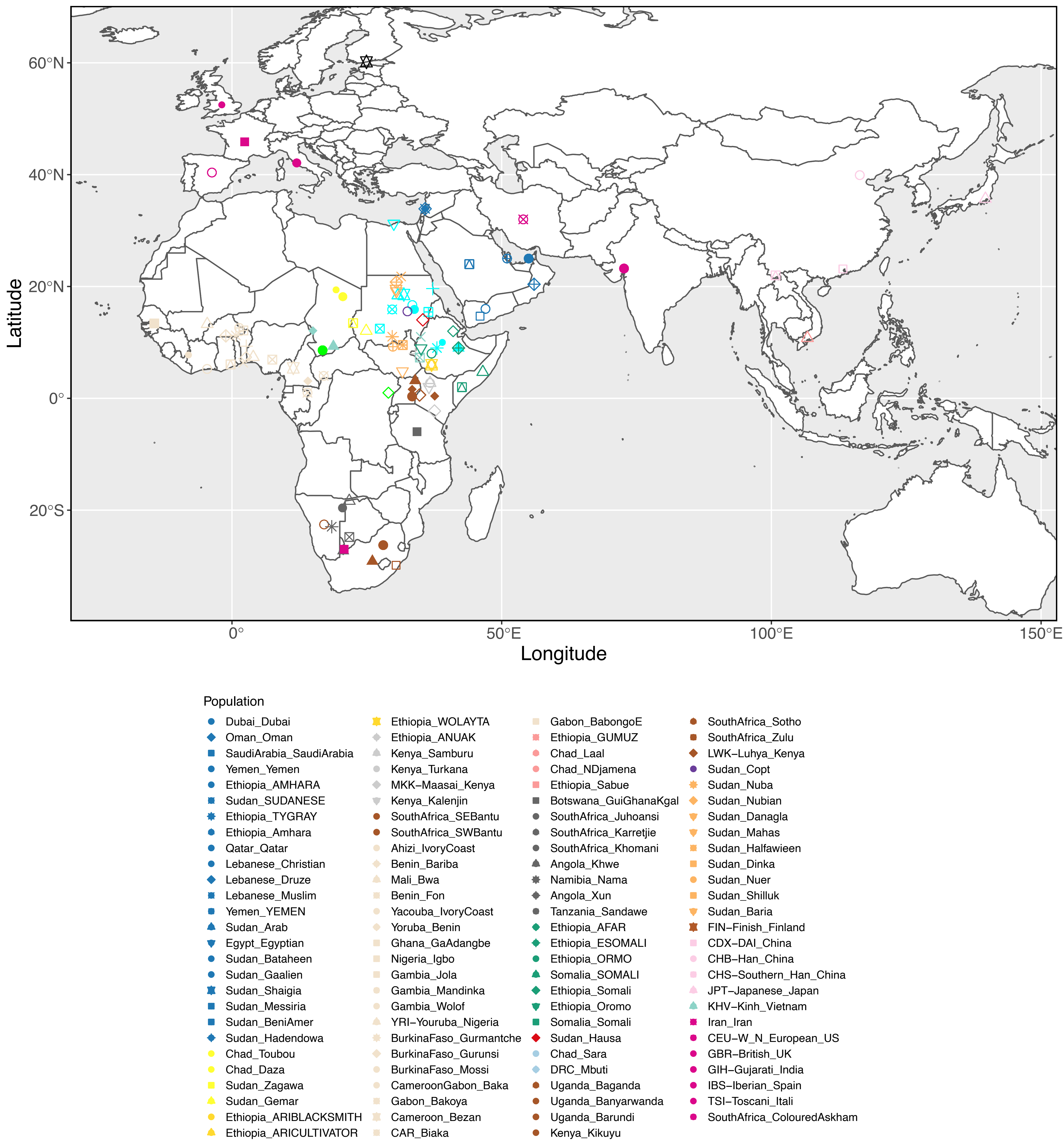

60°N
40°N
20°N
0°
20°S
Latitude
0°
50°E
100°E
150°E
Longitude
Population
Dubai_Dubai
Oman_Oman
SaudiArabia_SaudiArabia
Yemen_Yemen
Ethiopia_AMHARA
Sudan_SUDANESE
Ethiopia_TYGRAY
Ethiopia_Amhara
Qatar_Qatar
Lebanese_Christian
Lebanese_Druze
Lebanese_Muslim
Yemen_YEMEN
Sudan_Arab
Egypt_Egyptian
Sudan_Bataheen
Sudan_Gaalien
Sudan_Shaigia
Sudan_Messiria
Sudan_BeniAmer
Sudan_Hadendowa
Chad_Toubou
Chad_Daza
Sudan_Zagawa
Sudan_Gemar
Ethiopia_ARIBLACKSMITH
Ethiopia_ARICULTIVATOR
Ethiopia_WOLAYTA
Ethiopia_ANUAK
Kenya_Samburu
Kenya_Turkana
MKK–Maasai_Kenya
Kenya_Kalenjin
SouthAfrica_SEBantu
SouthAfrica_SWBantu
Ahizi_IvoryCoast
Benin_Bariba
Mali_Bwa
Benin_Fon
Yacouba_IvoryCoast
Yoruba_Benin
Ghana_GaAdangbe
Nigeria_Igbo
Gambia_Jola
Gambia_Mandinka
Gambia_Wolof
YRI–Youruba_Nigeria
BurkinaFaso_Gurmantche
BurkinaFaso_Gurunsi
BurkinaFaso_Mossi
CameroonGabon_Baka
Gabon_Bakoya
Cameroon_Bezan
CAR_Biaka
Gabon_BabongoE
Ethiopia_GUMUZ
Chad_Laal
Chad_NDjamena
Ethiopia_Sabue
Botswana_GuiGhanaKgal
SouthAfrica_Juhoansi
SouthAfrica_Karretjie
SouthAfrica_Khomani
Angola_Khwe
Namibia_Nama
Angola_Xun
Tanzania_Sandawe
Ethiopia_AFAR
Ethiopia_ESOMALI
Ethiopia_ORMO
Somalia_SOMALI
Ethiopia_Somali
Ethiopia_Oromo
Somalia_Somali
Sudan_Hausa
Chad_Sara
DRC_Mbuti
Uganda_Baganda
Uganda_Banyarwanda
Uganda_Barundi
Kenya_Kikuyu
SouthAfrica_Sotho
SouthAfrica_Zulu
LWK–Luhya_Kenya
Sudan_Copt
Sudan_Nuba
Sudan_Nubian
Sudan_Danagla
Sudan_Mahas
Sudan_Halfawieen
Sudan_Dinka
Sudan_Nuer
Sudan_Shilluk
Sudan_Baria
FIN–Finish_Finland
CDX–DAI_China
CHB–Han_China
CHS–Southern_Han_China
JPT–Japanese_Japan
KHV–Kinh_Vietnam
Iran_Iran
CEU–W_N_European_US
GBR–British_UK
GIH–Gujarati_India
IBS–Iberian_Spain
TSI–Toscani_Itali
SouthAfrica_ColouredAskham
