# Supplementary figures and images for: Eurasian back-migration into Northeast Africa was a complex and multifaceted process
Source: PLoS One. 2023 Nov 8;18(11):e0290423. doi: 10.1371/journal.pone.0290423 (PMC10631636; doi:10.1371/journal.pone.0290423)

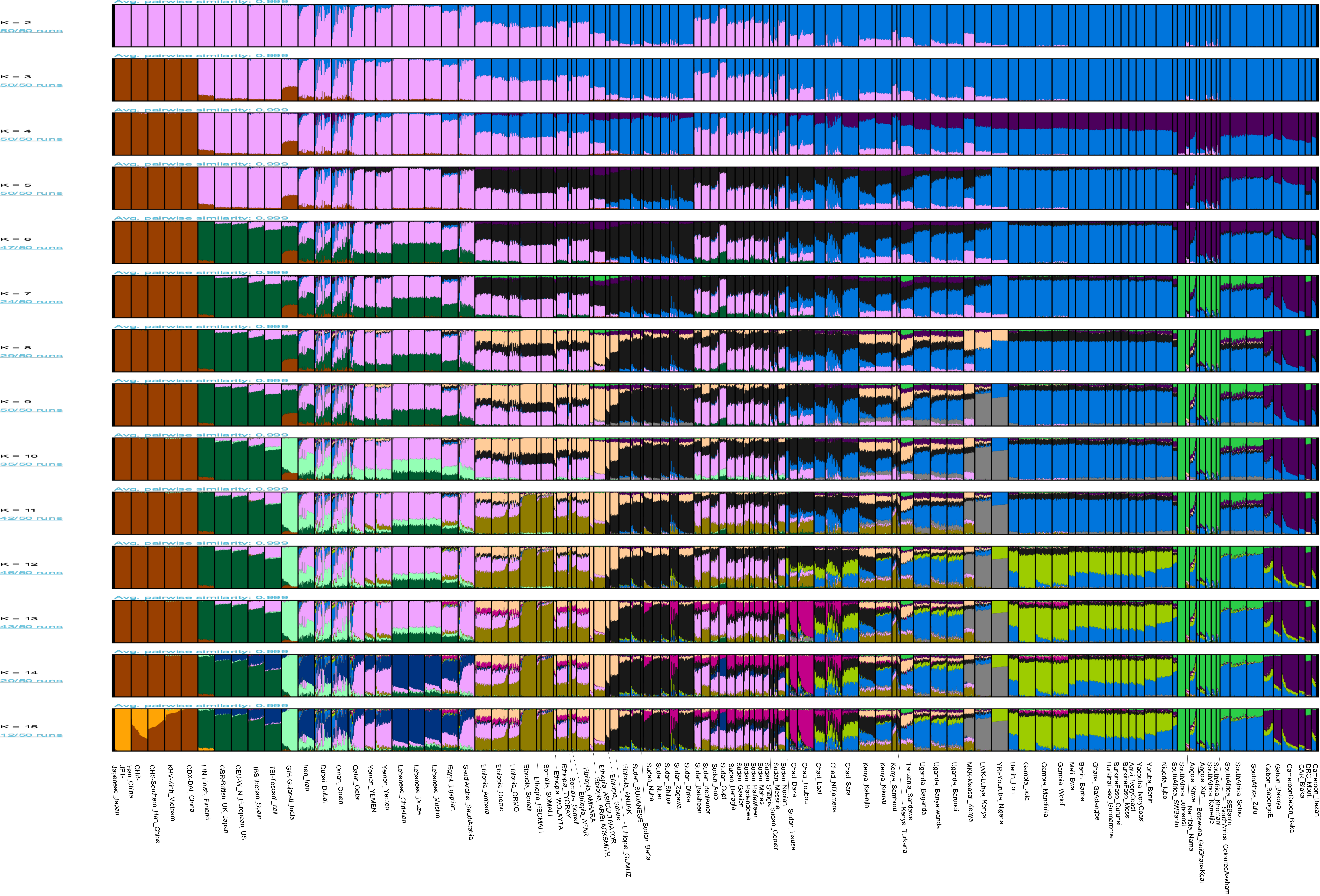

Supplement: S2 Fig — 50 iterations for the full dataset. The best identified K through cross-validation was K = 13. (PDF) [file pone.0290423.s008.pdf]

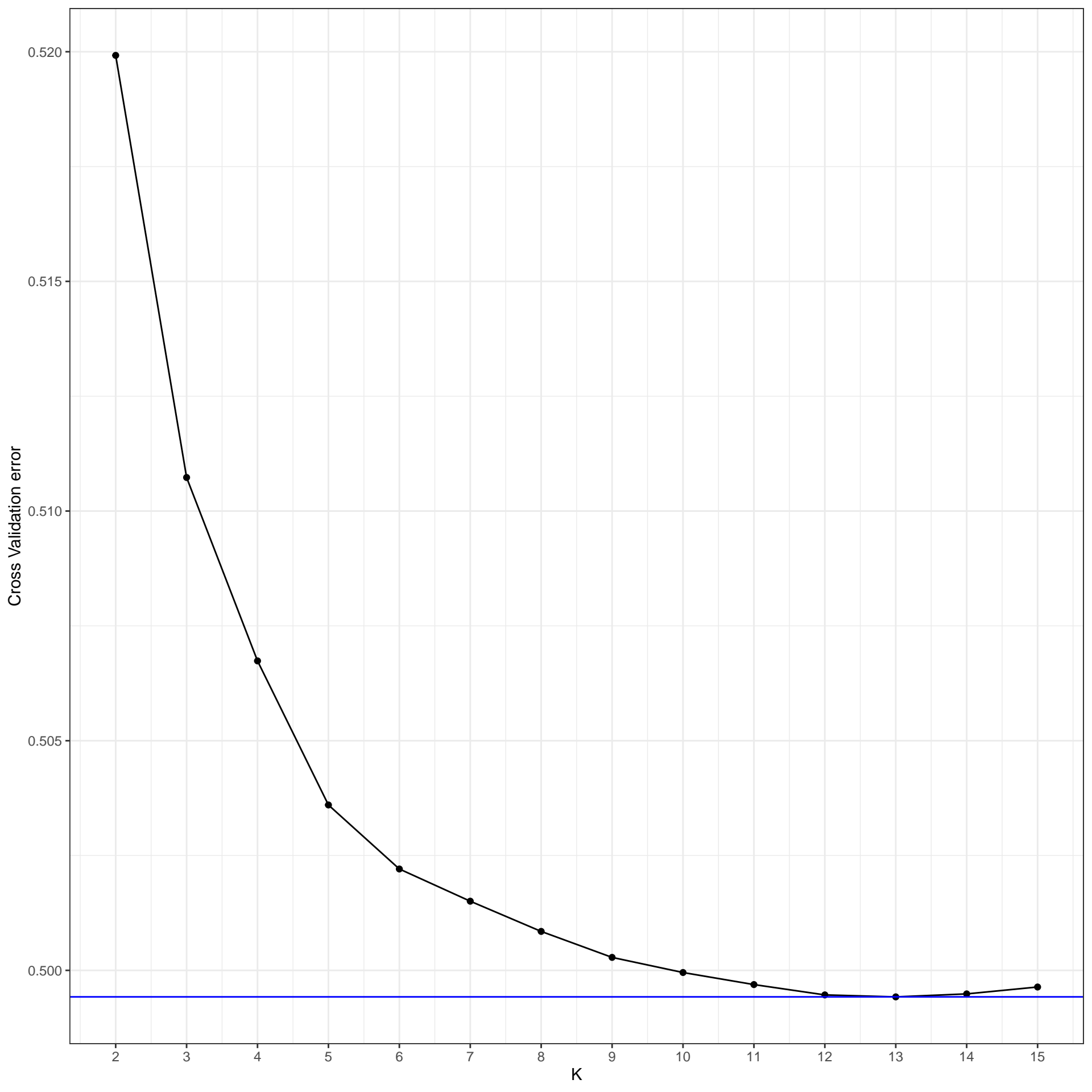

Supplement: S3 Fig — The K with the lowest CV error was K = 13, indicated by the horizontal line. (PDF) [file pone.0290423.s009.pdf]

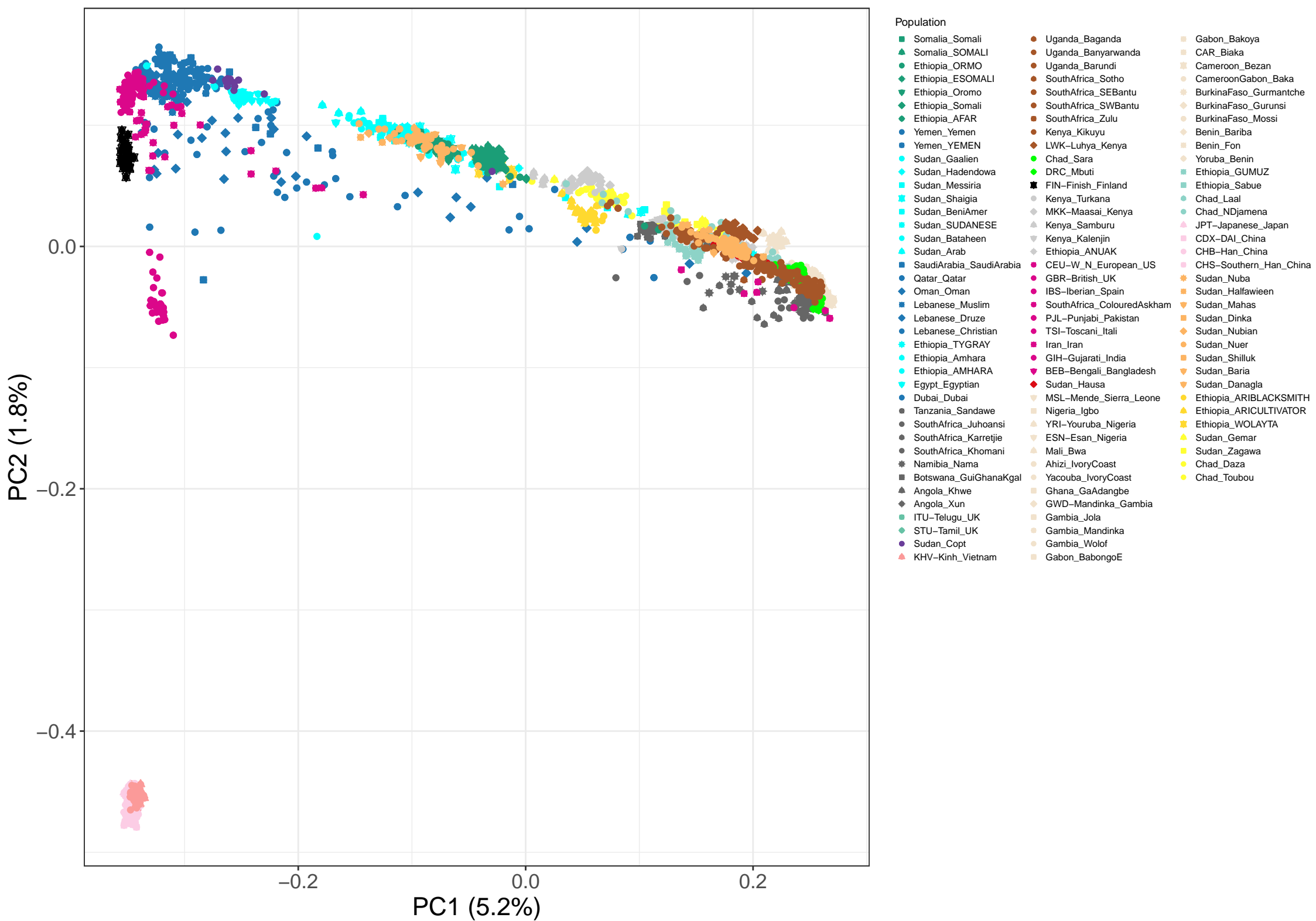

Supplement: S5 Fig — Values within parenthesis are the PC loading. Populations are coloured by linguistic group. (PDF) [file pone.0290423.s011.pdf]

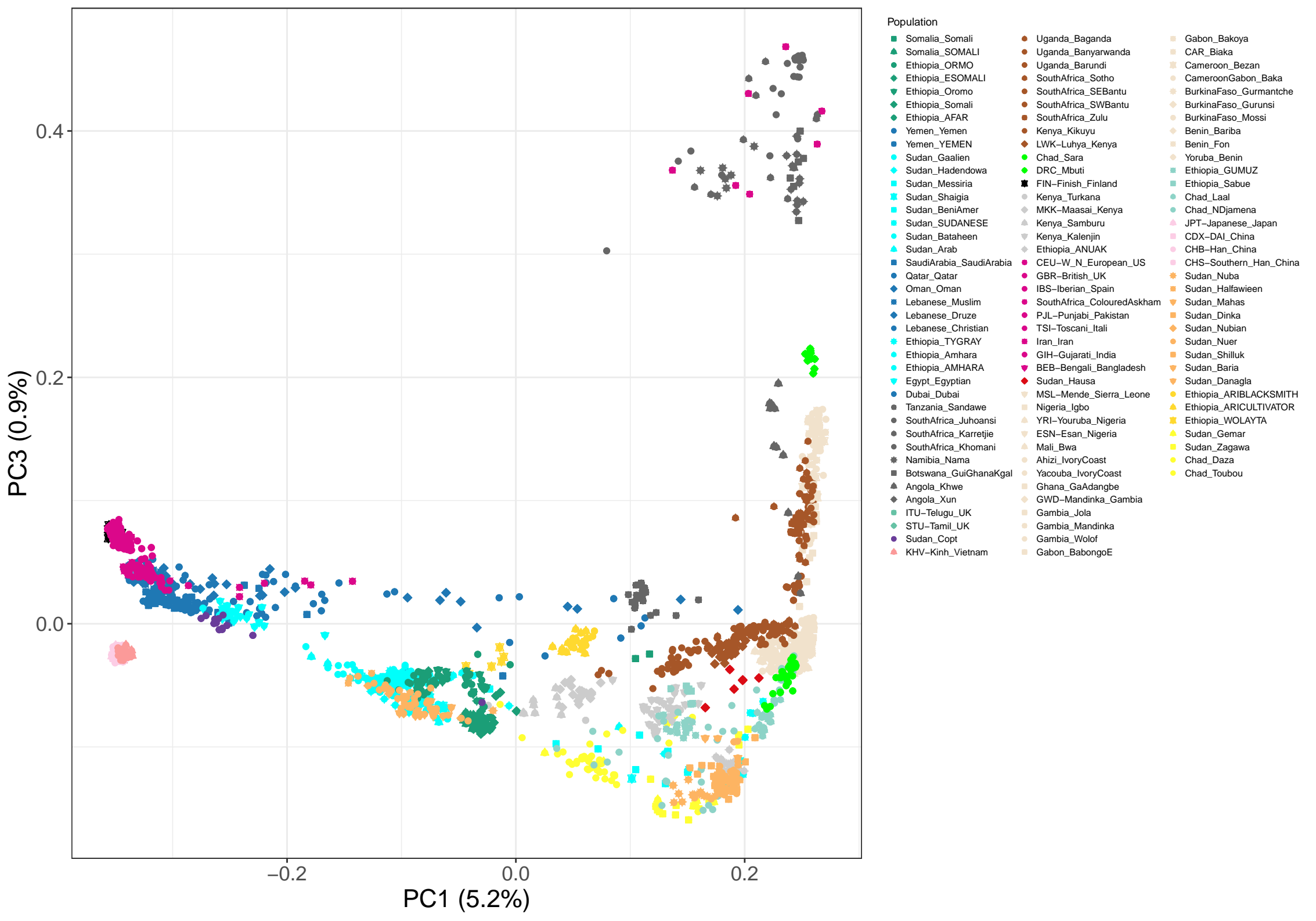

Supplement: S6 Fig — (PDF) [file pone.0290423.s012.pdf]

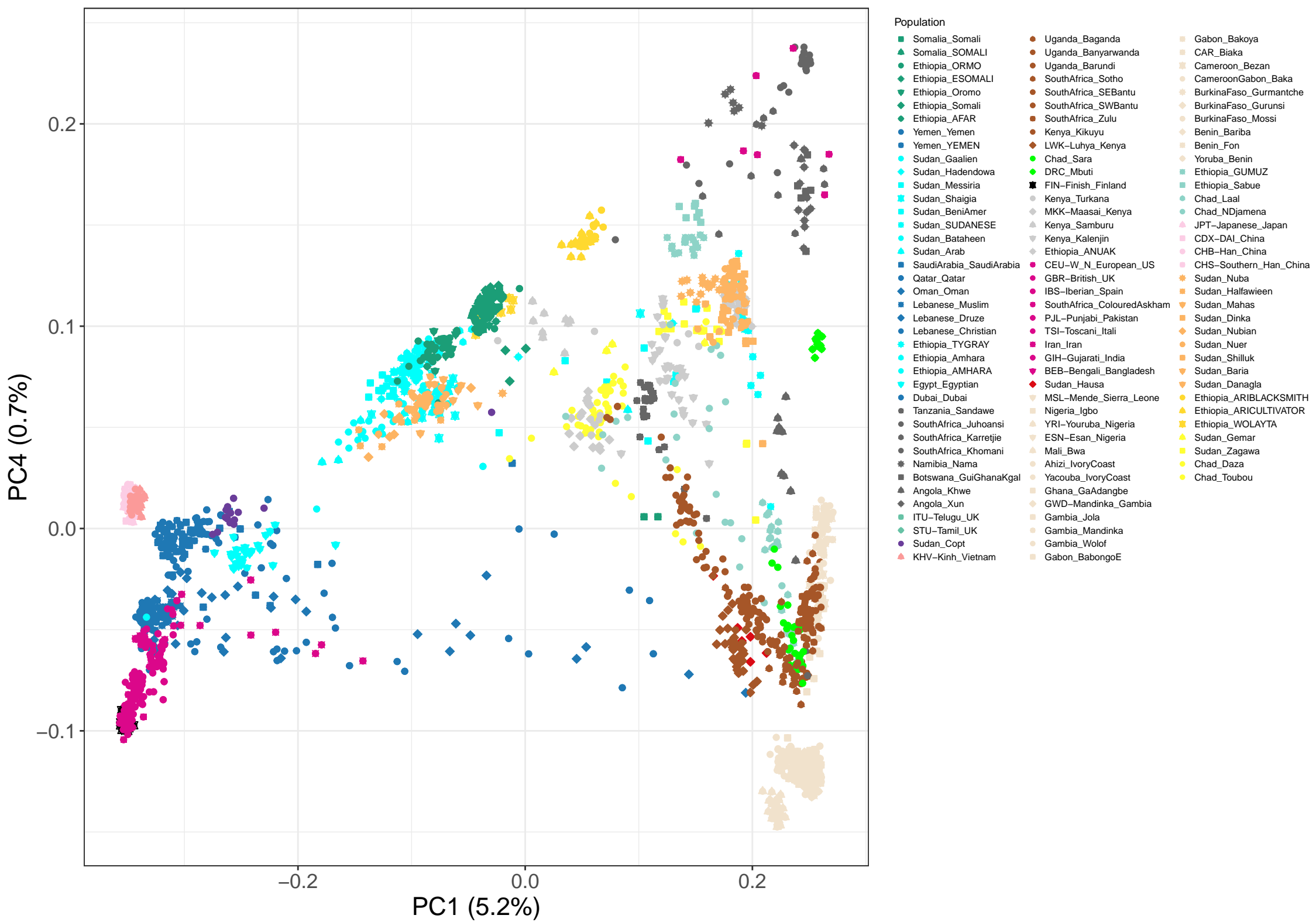

Supplement: S7 Fig — (PDF) [file pone.0290423.s013.pdf]

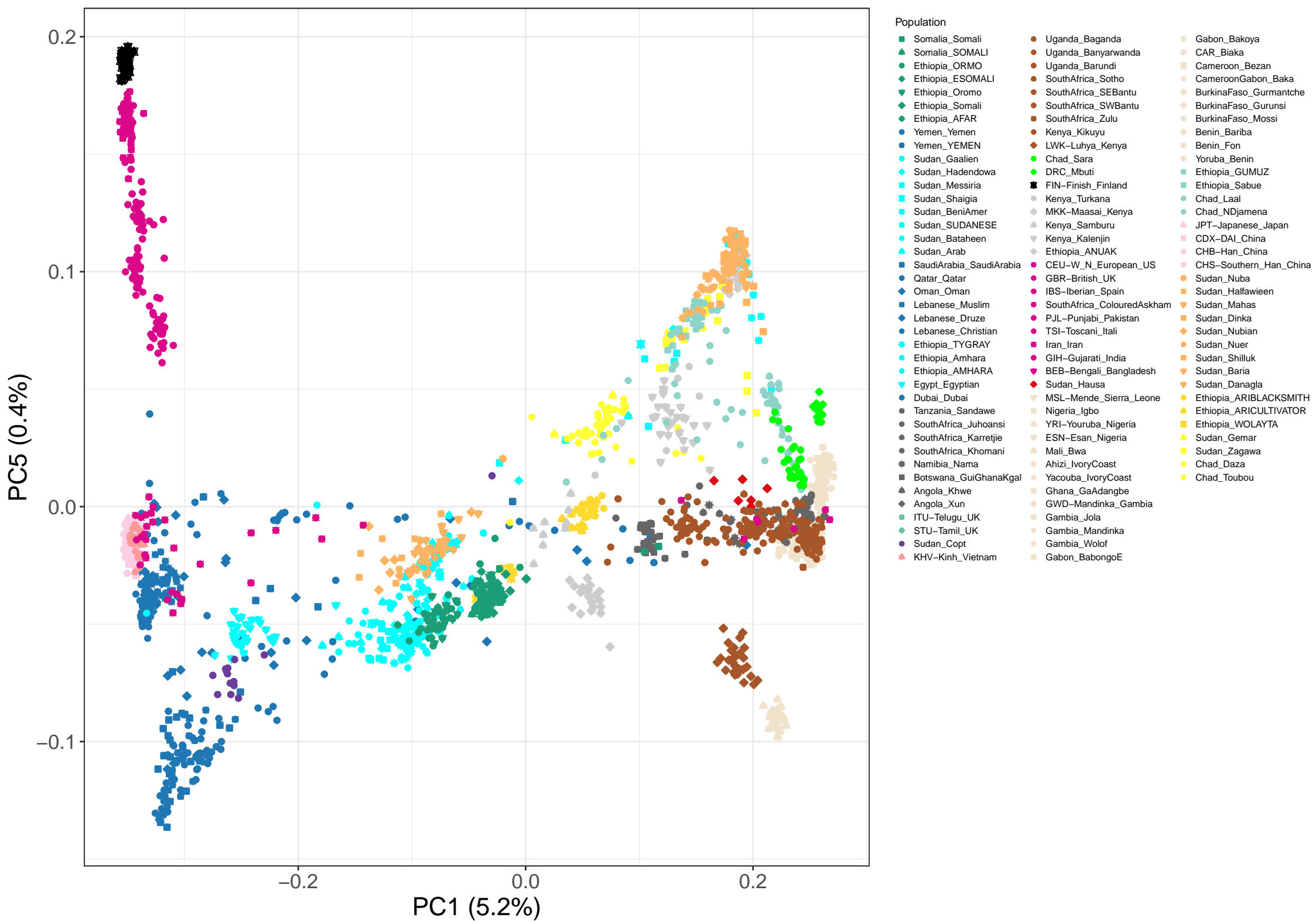

Supplement: S8 Fig — (PDF) [file pone.0290423.s014.pdf]

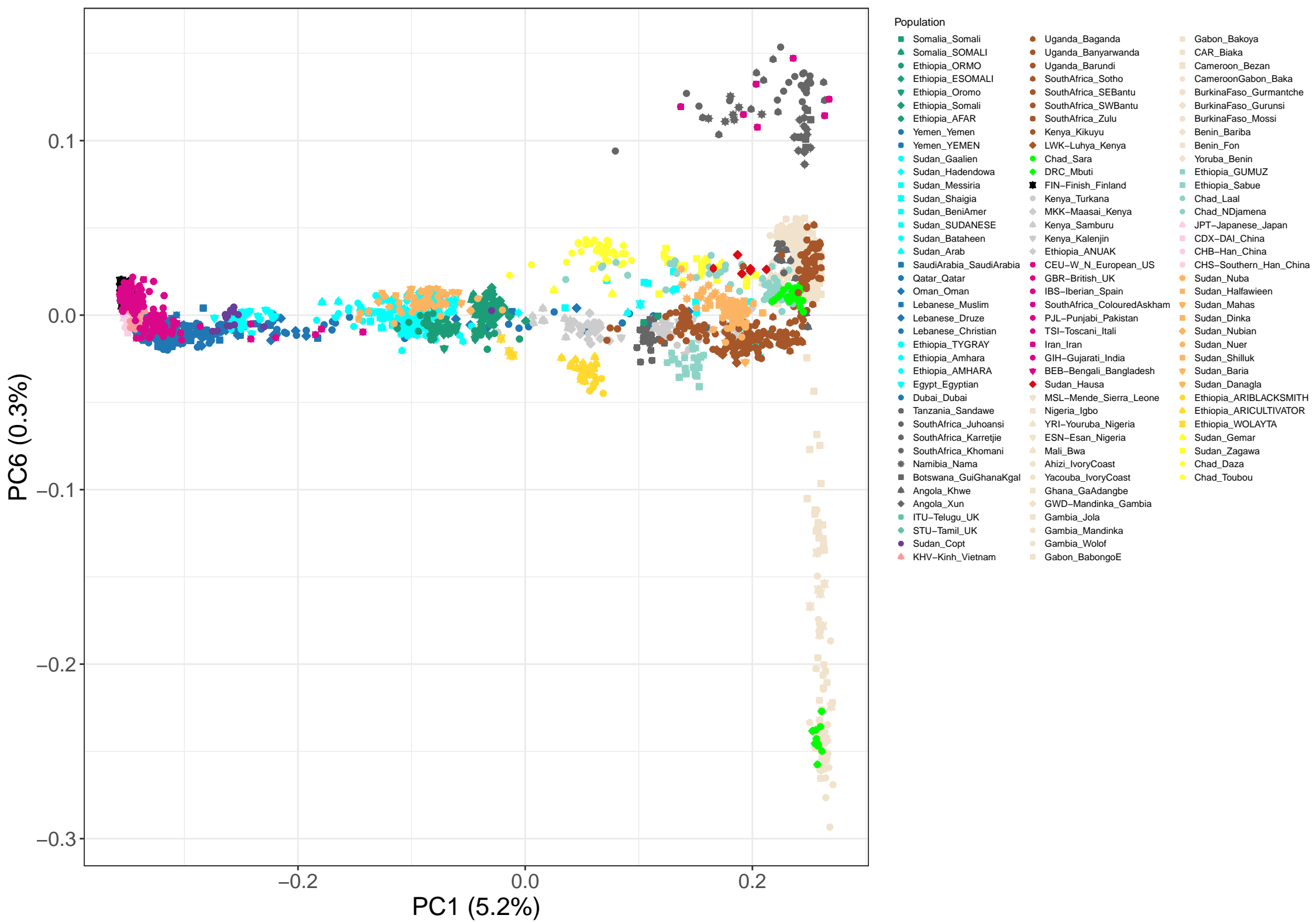

Supplement: S9 Fig — (PDF) [file pone.0290423.s015.pdf]

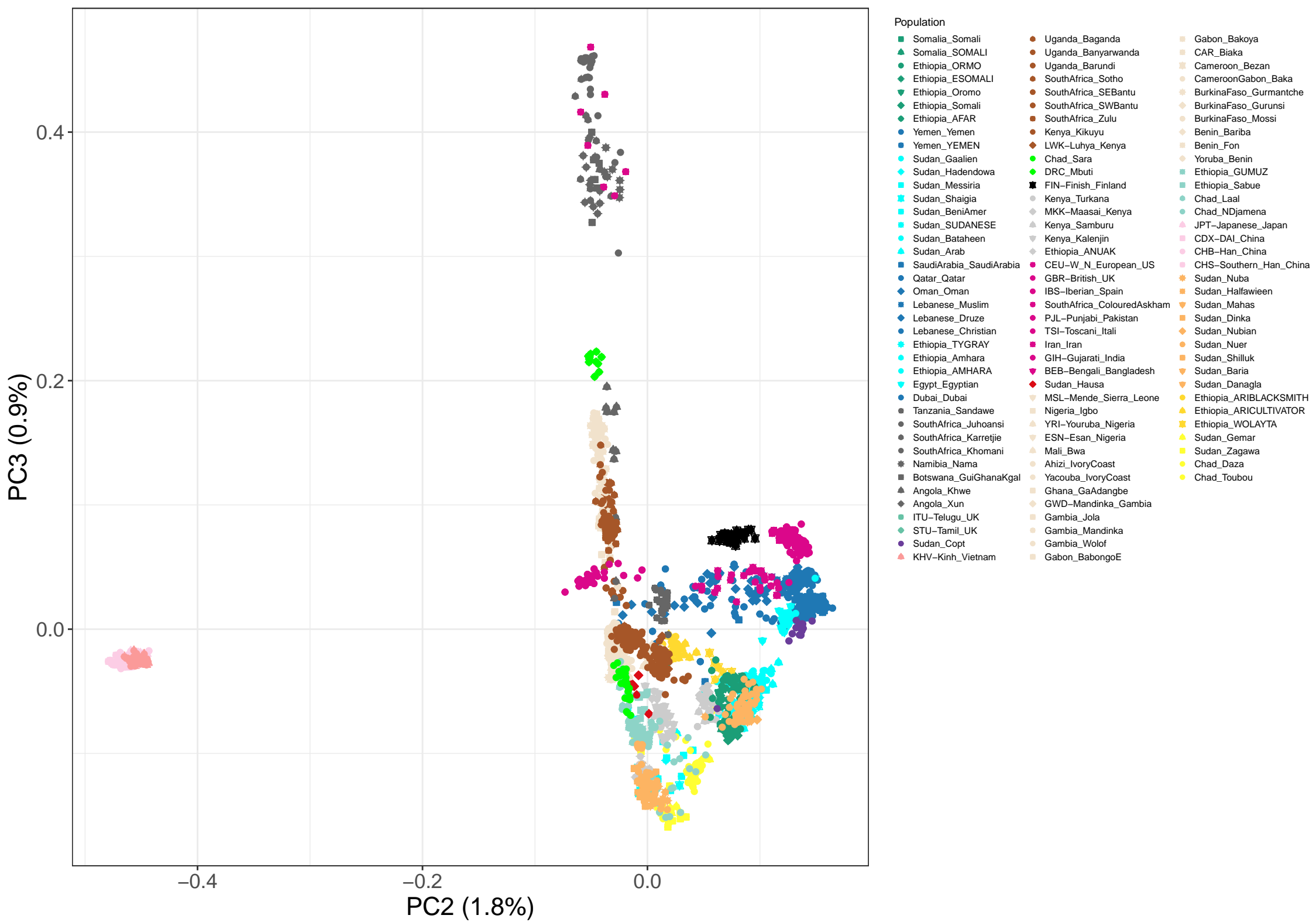

Supplement: S10 Fig — (PDF) [file pone.0290423.s016.pdf]

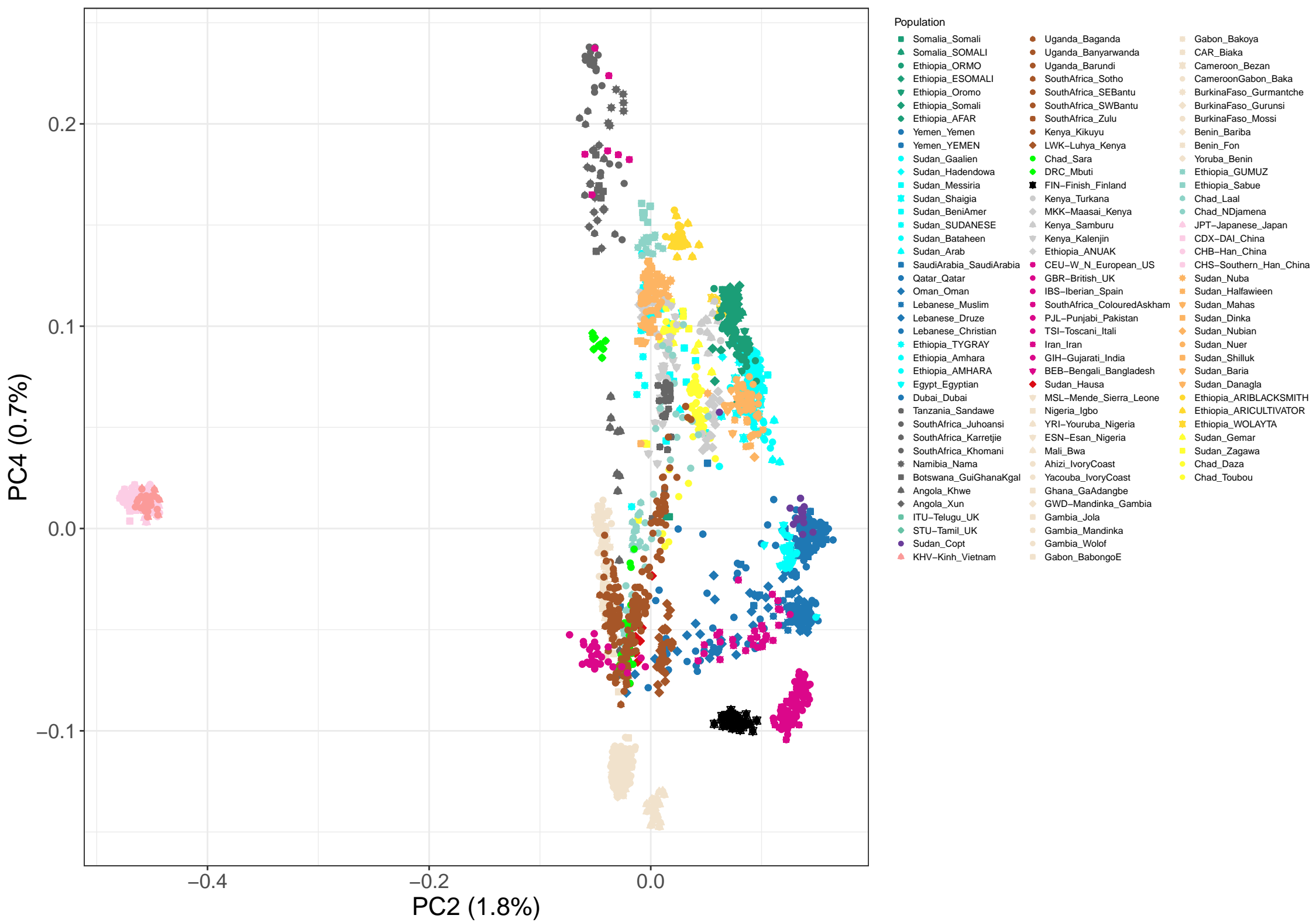

Supplement: S11 Fig — (PDF) [file pone.0290423.s017.pdf]

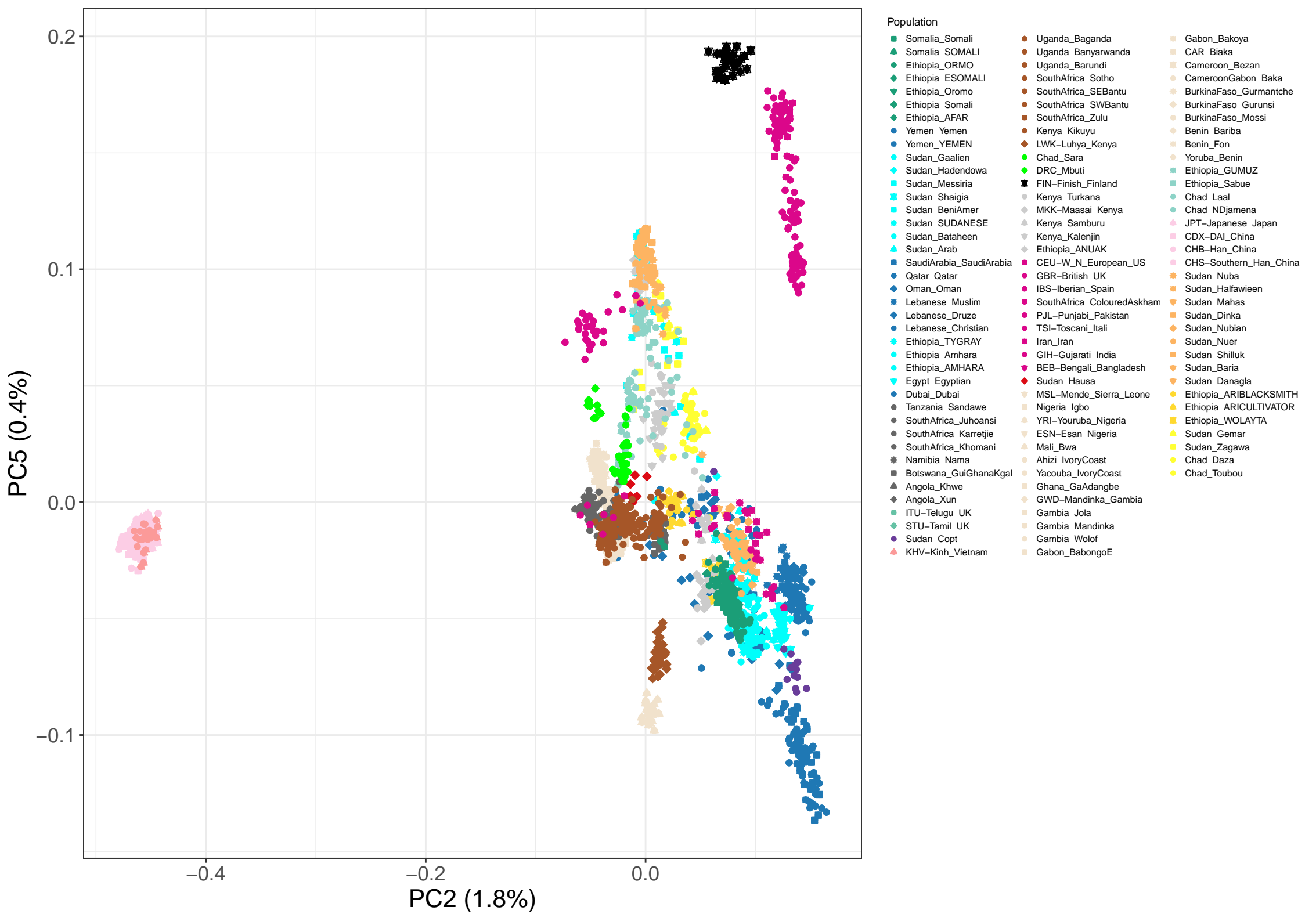

Supplement: S12 Fig — (PDF) [file pone.0290423.s018.pdf]

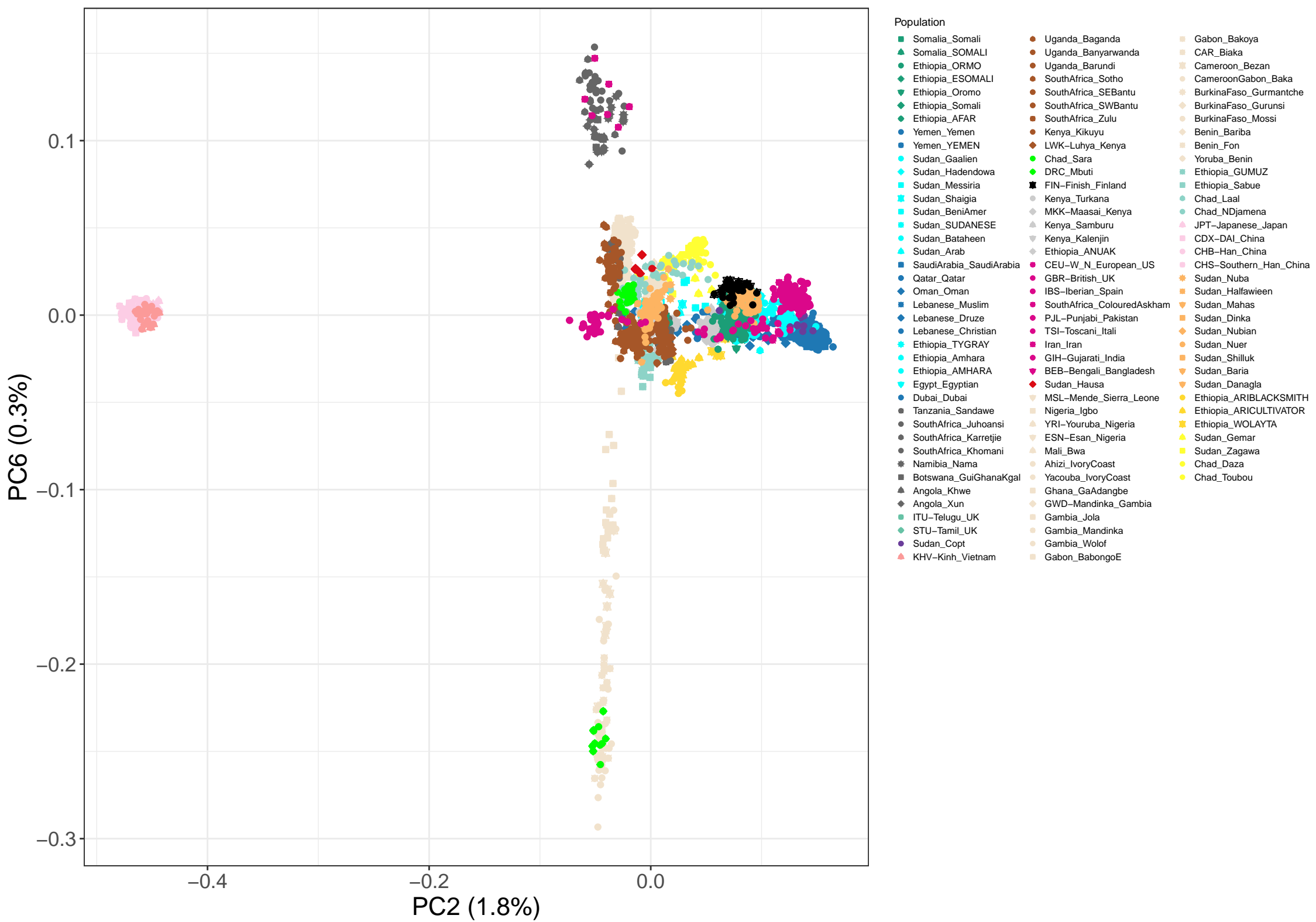

Supplement: S13 Fig — (PDF) [file pone.0290423.s019.pdf]

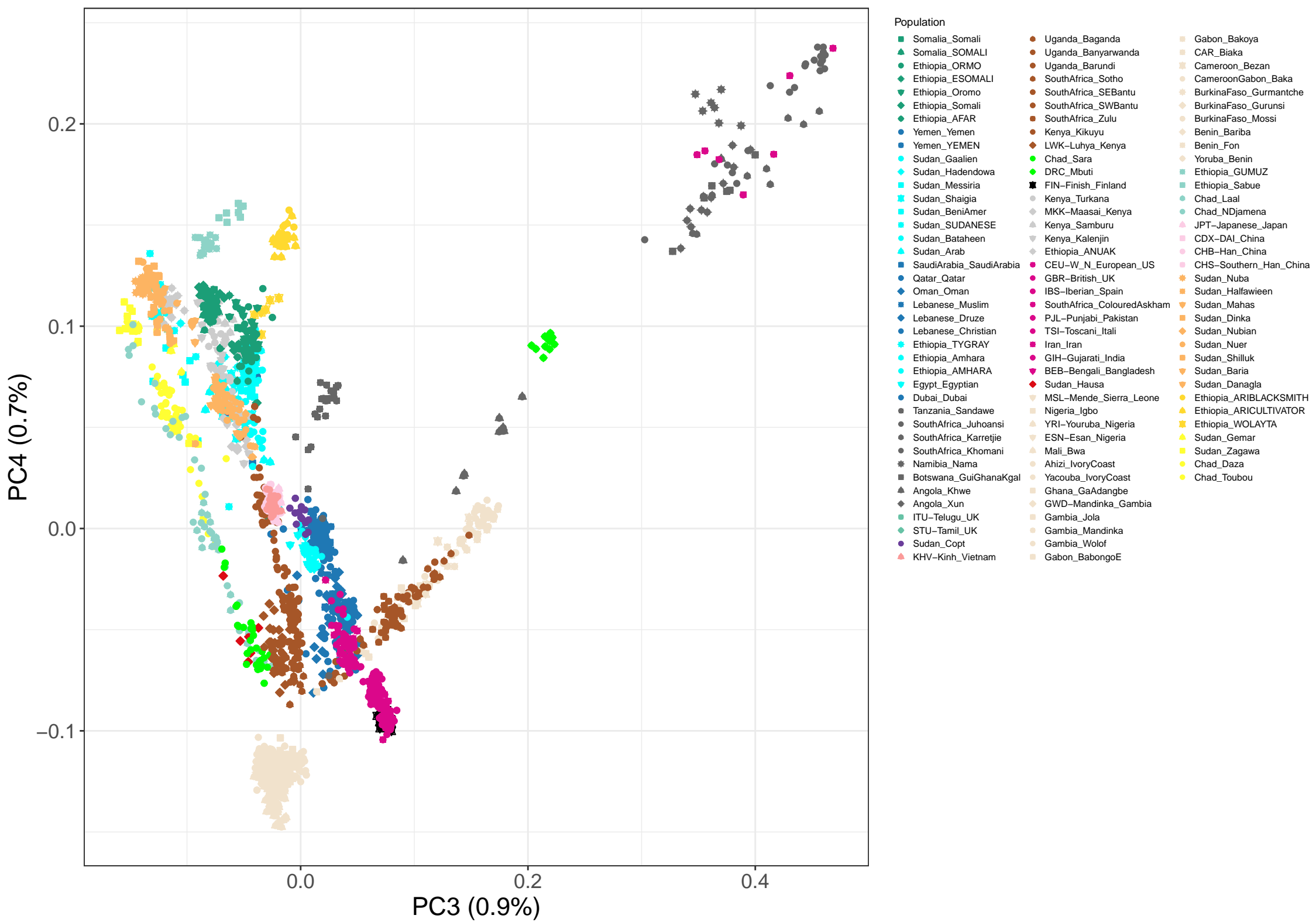

Supplement: S14 Fig — (PDF) [file pone.0290423.s020.pdf]

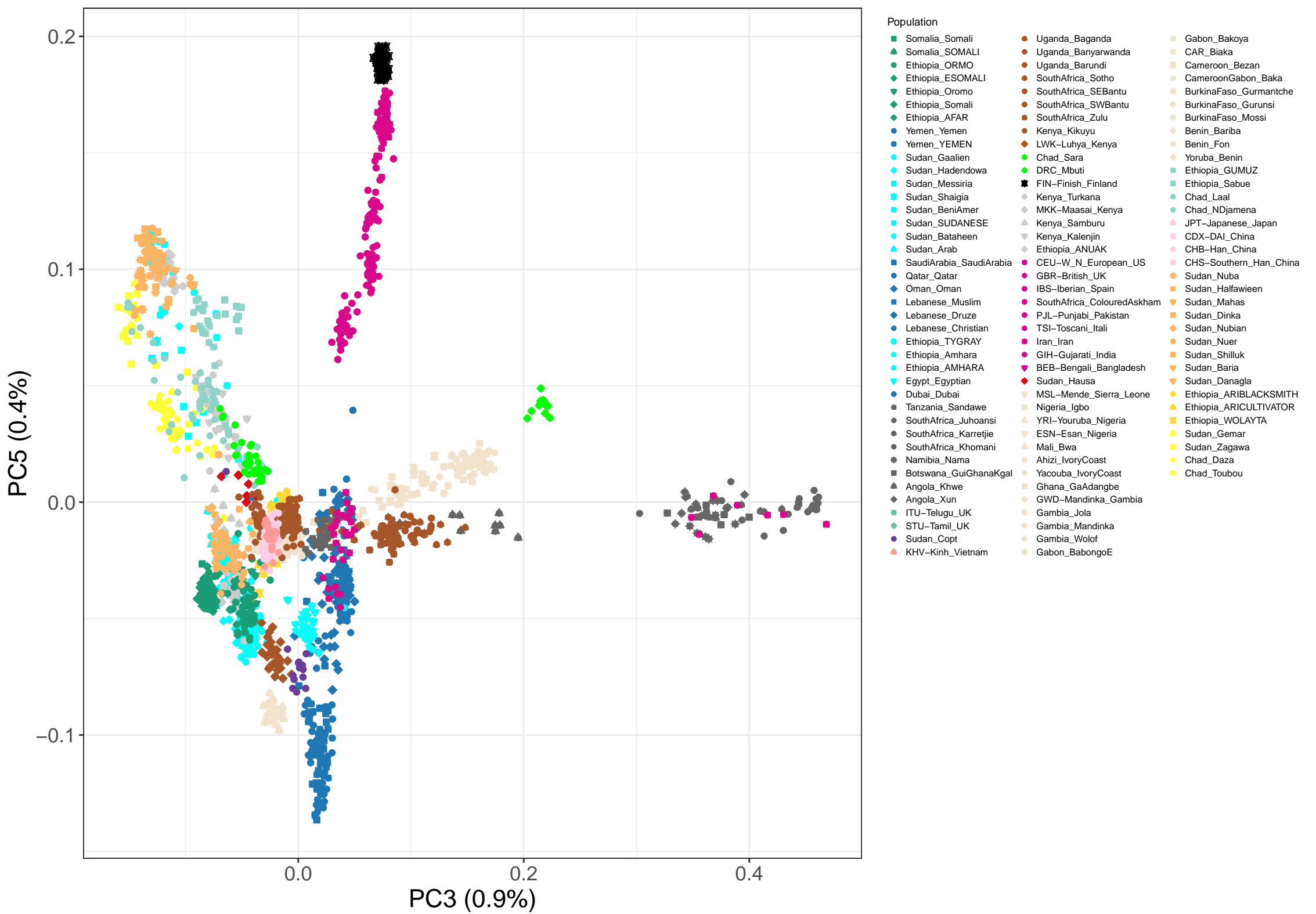

Supplement: S15 Fig — (PDF) [file pone.0290423.s021.pdf]

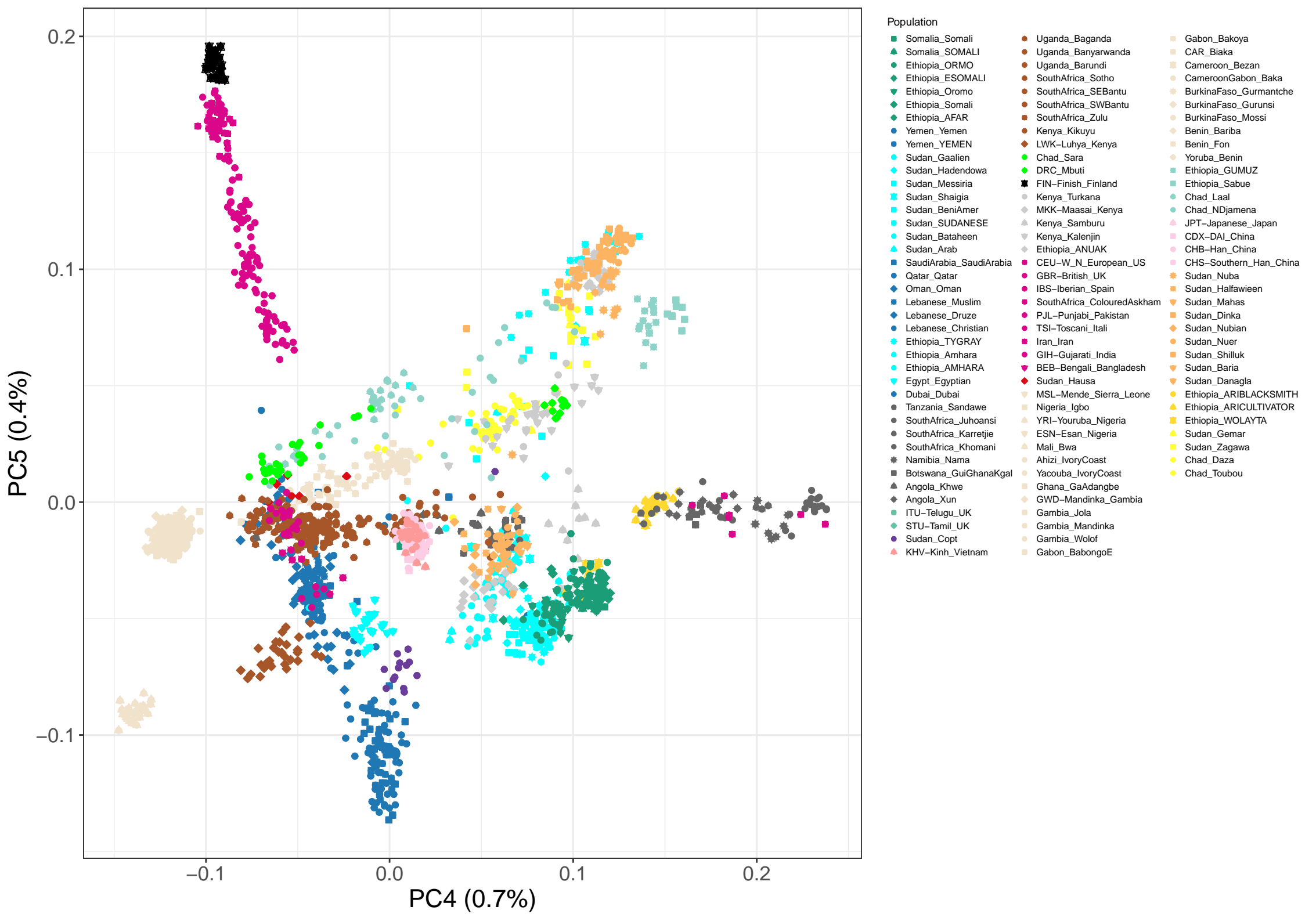

Supplement: S17 Fig — (PDF) [file pone.0290423.s023.pdf]

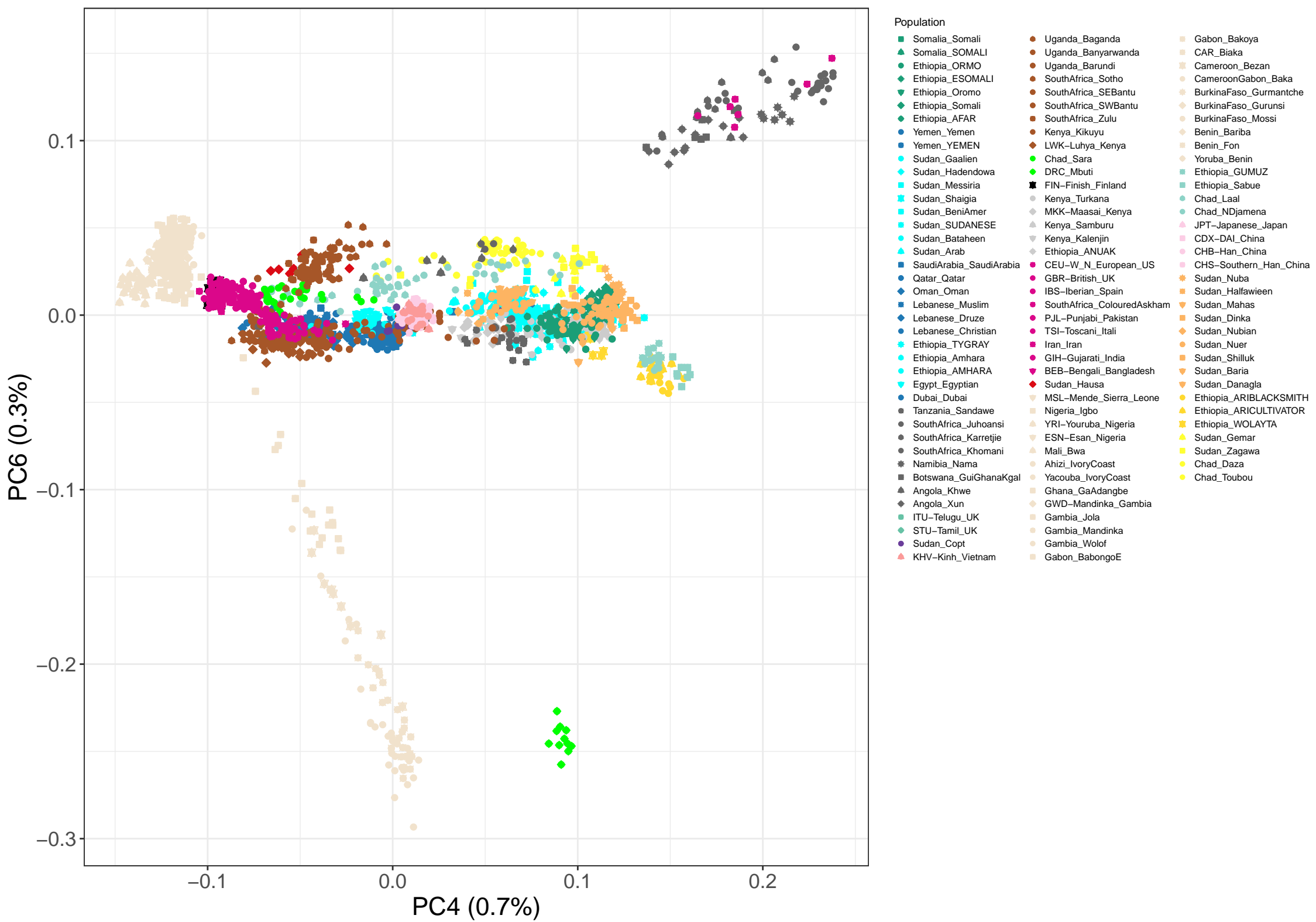

Supplement: S18 Fig — (PDF) [file pone.0290423.s024.pdf]

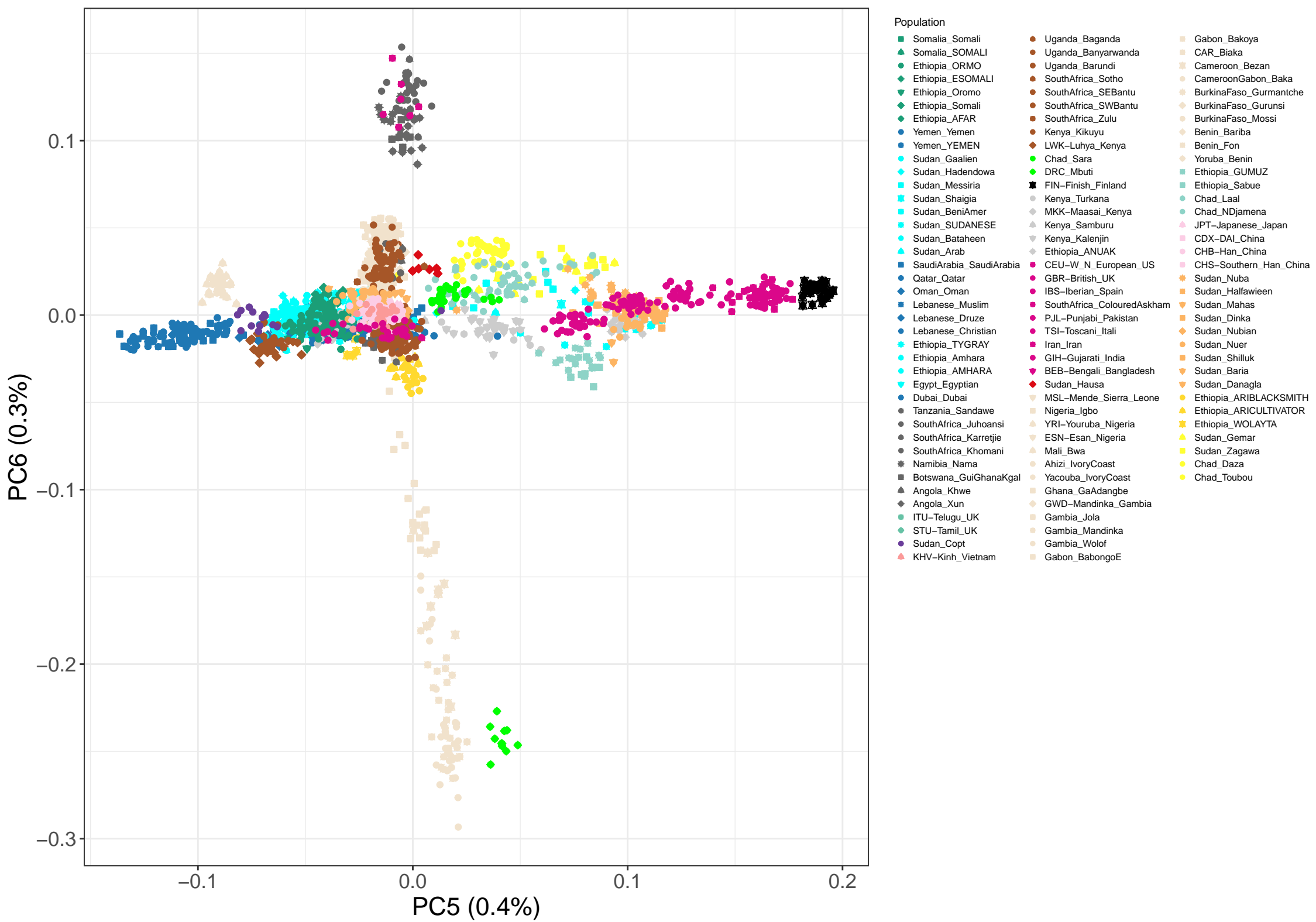

Supplement: S19 Fig — (PDF) [file pone.0290423.s025.pdf]

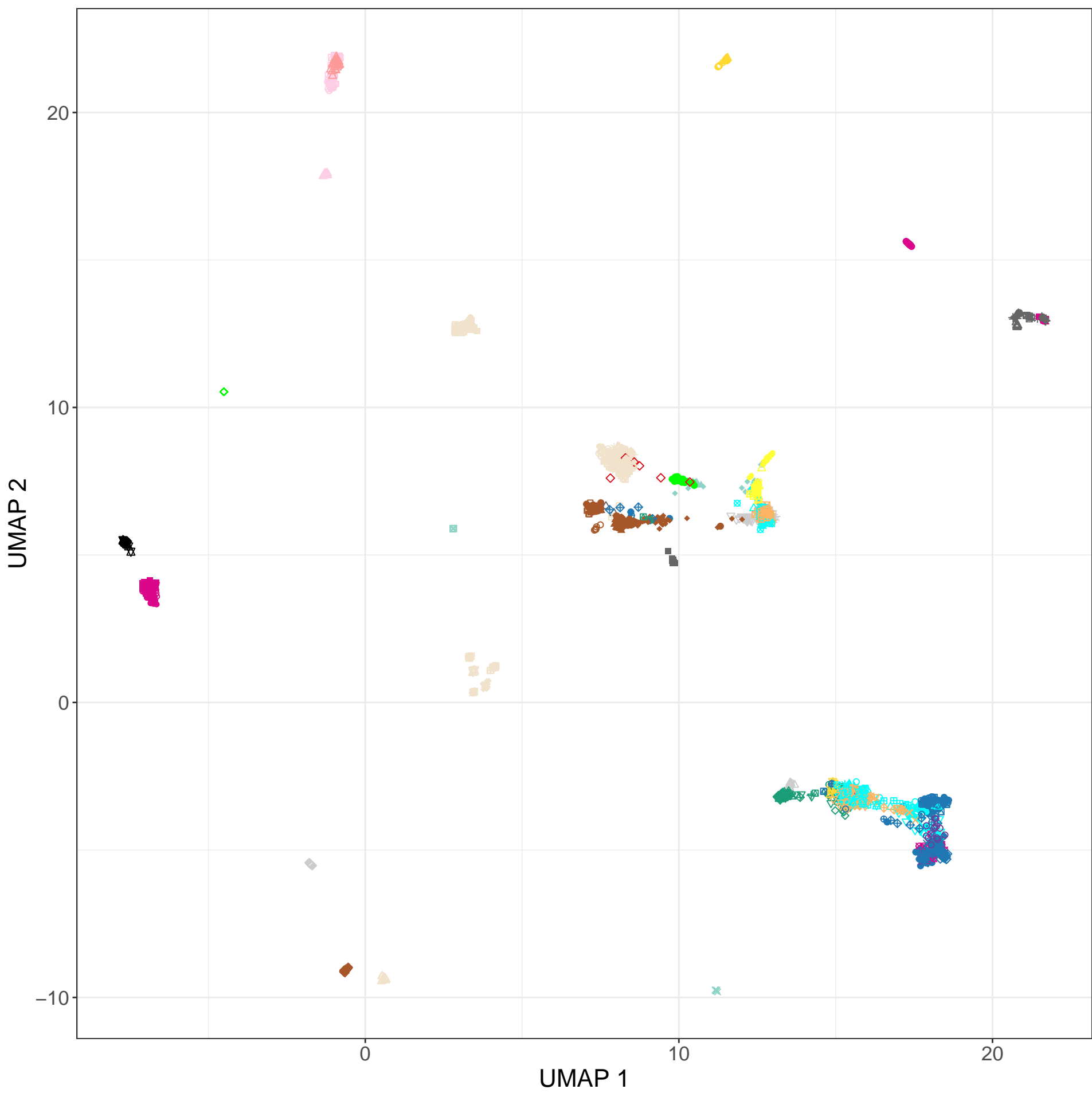

Supplement: S20 Fig — Colours are the same as in S1 Fig. (PDF) [file pone.0290423.s026.pdf]

**A**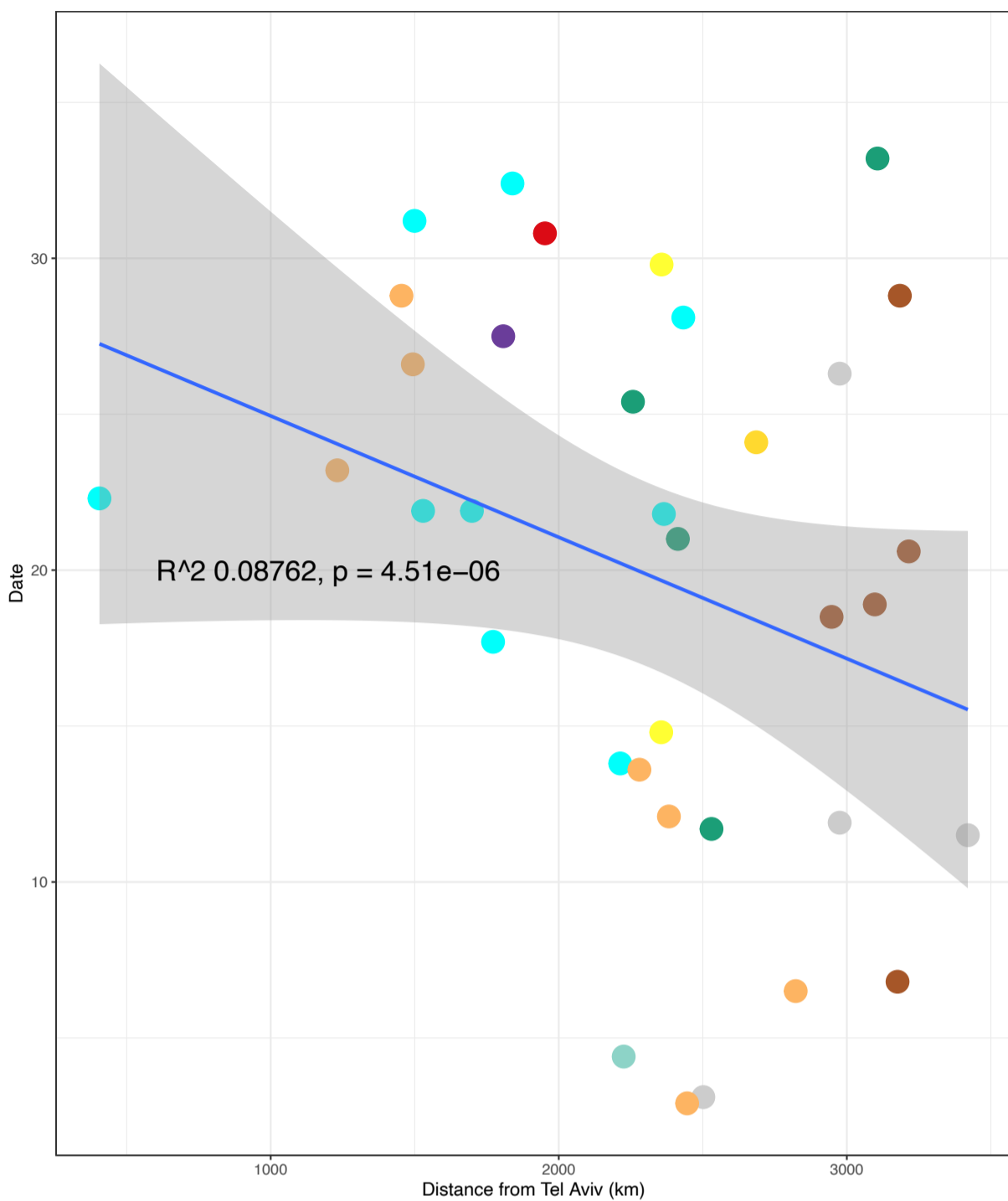**B**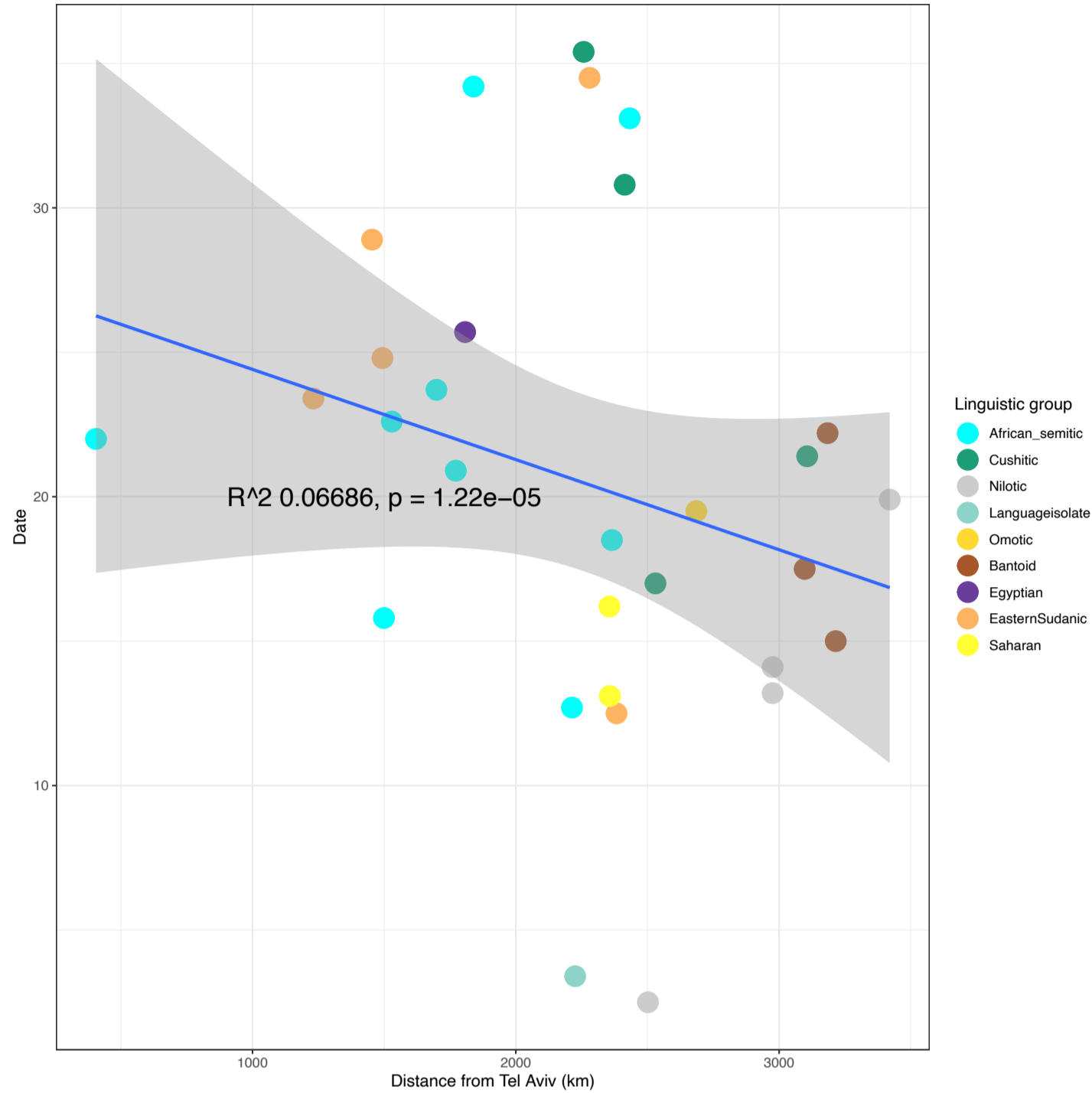**C**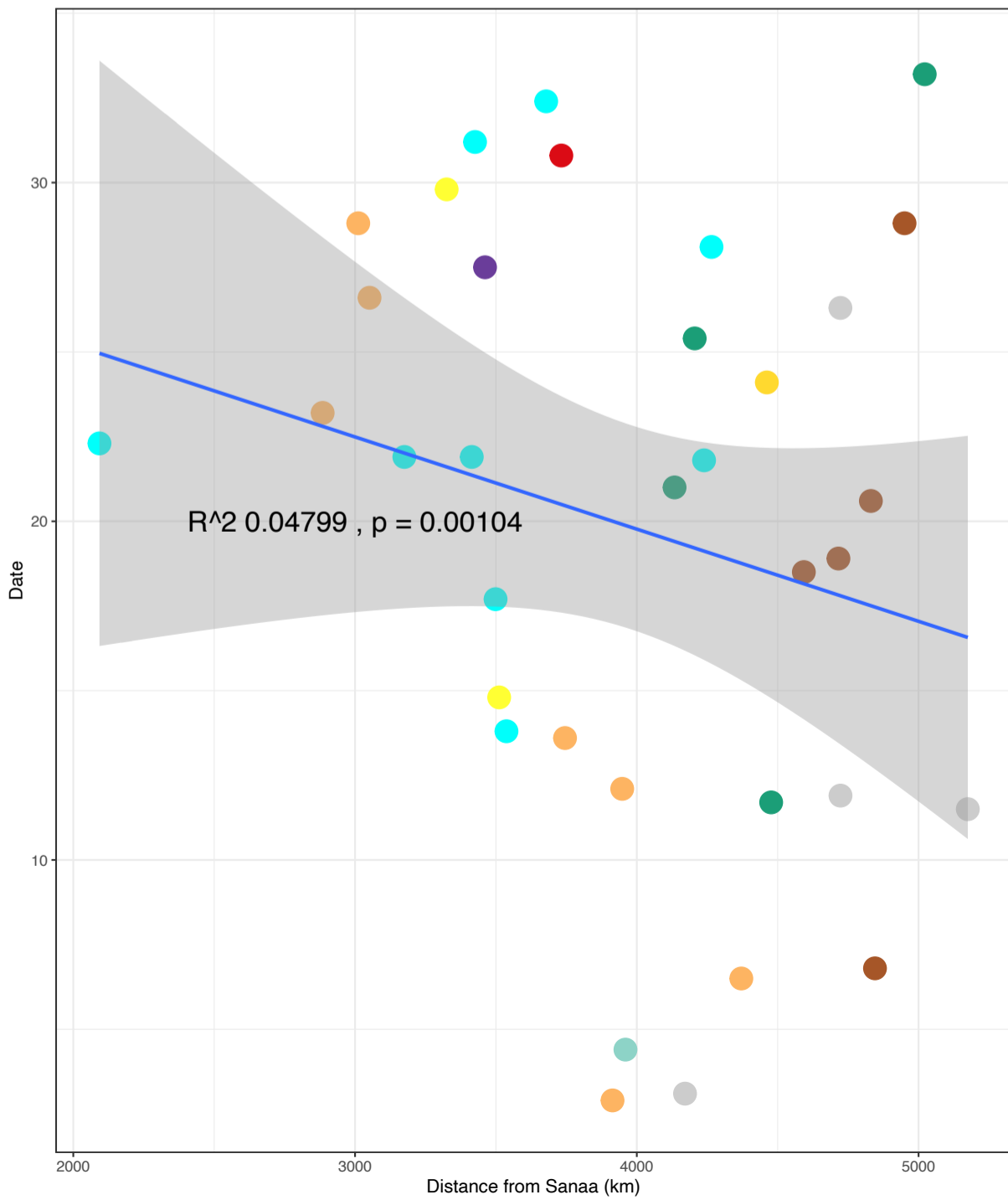**D**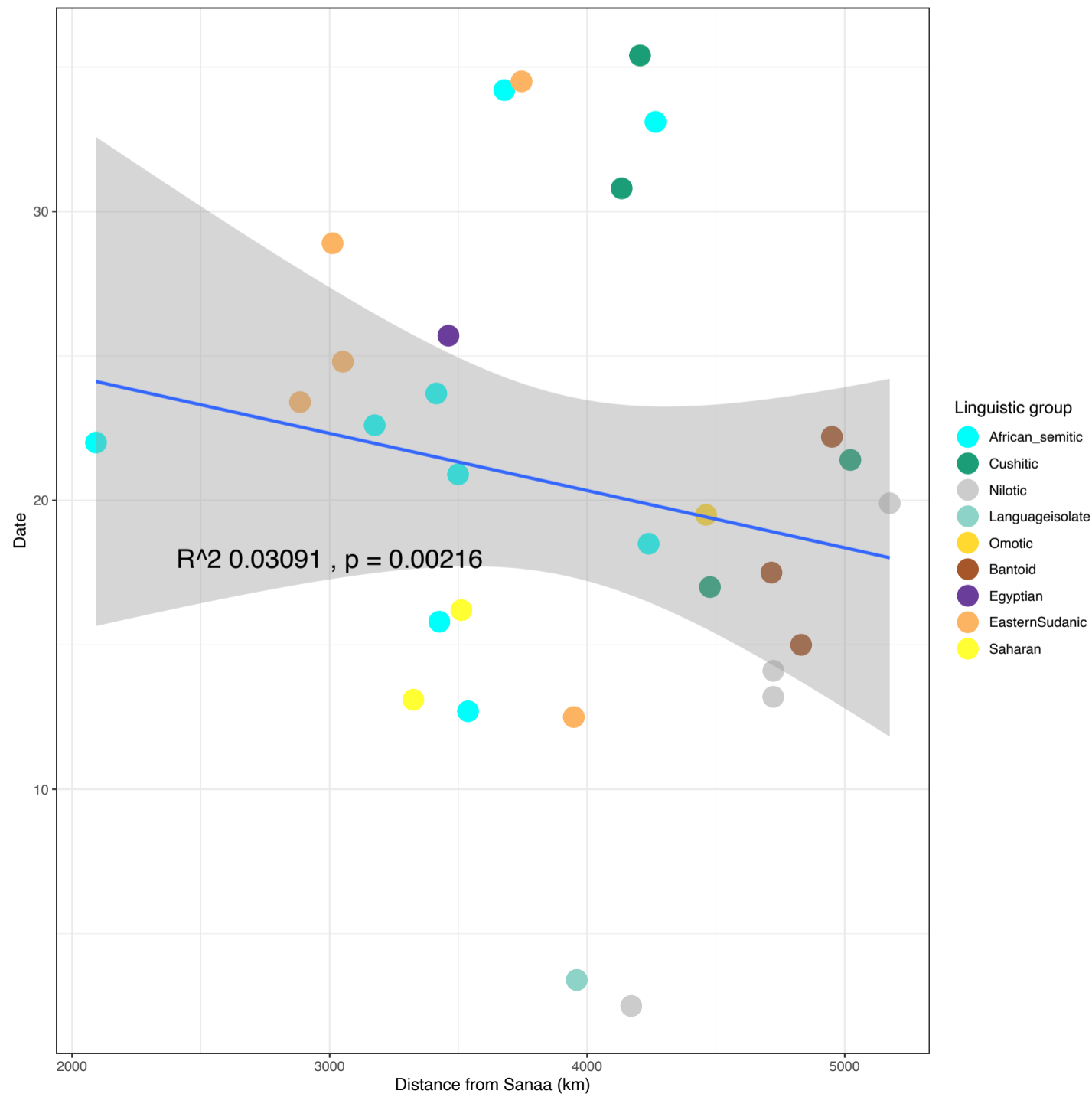

Supplement: S21 Fig — The blue line is the fitted linear regression line and the grey area represents the 95% confidence interval of the standard error. A) Distance from Tel Aviv for the best by f3 dataset. B) Distance from Tel Aviv for the best by R2 dataset. C) Distance from Sanaa for the best by f3 dataset. D) Distance from Sanaa for the best by R2. (PDF) [file pone.0290423.s027.pdf]

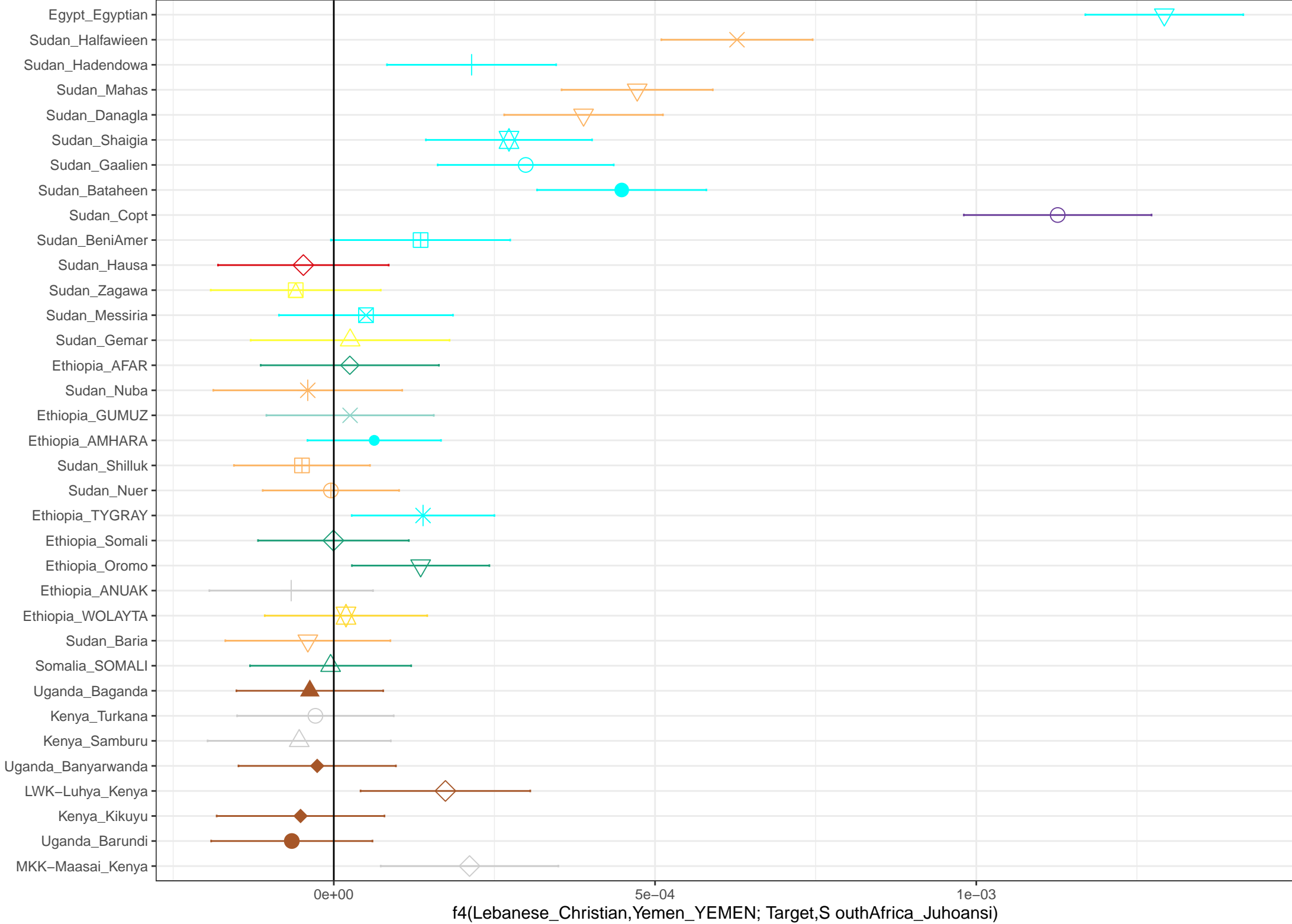

Supplement: S22 Fig — (PDF) [file pone.0290423.s028.pdf]

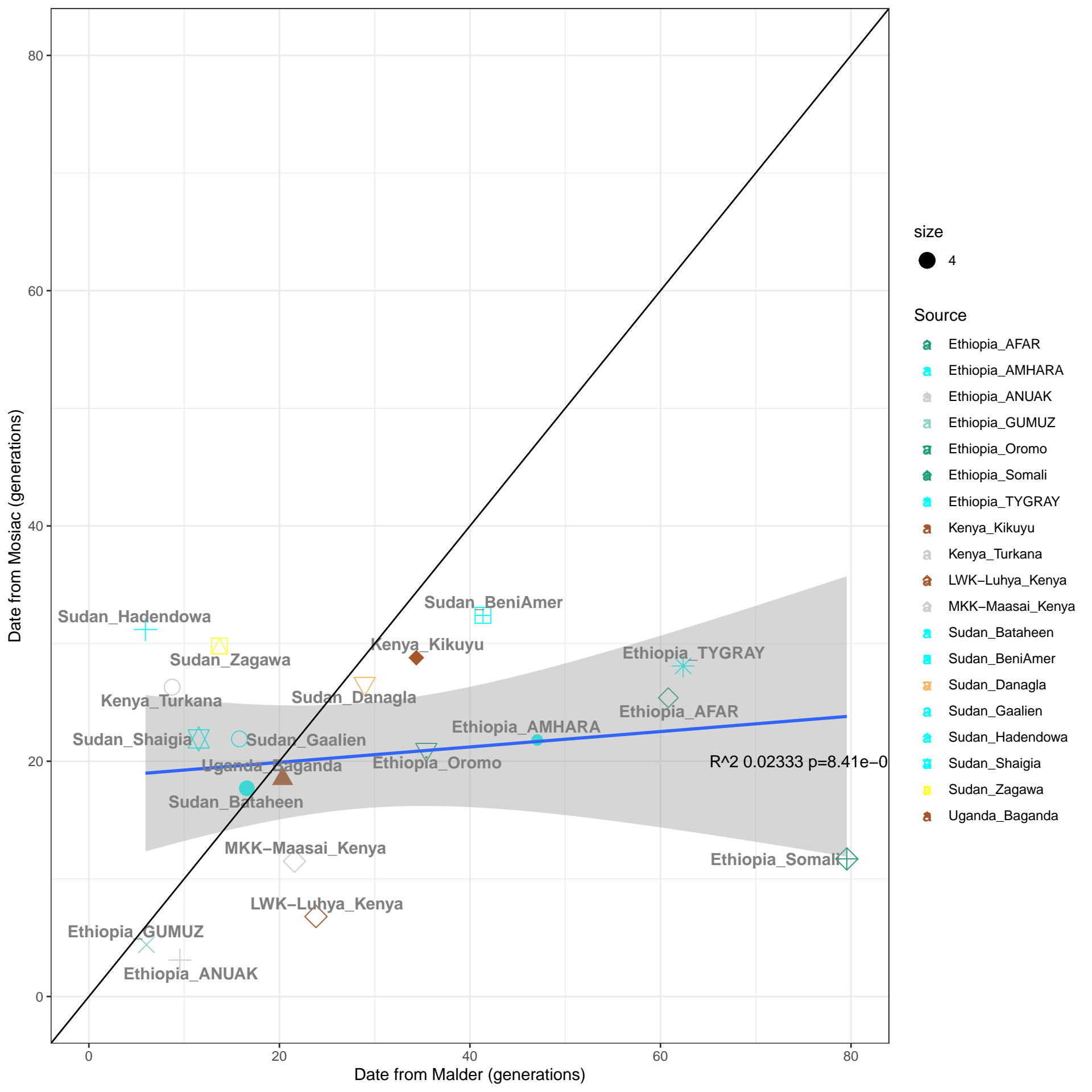

Supplement: S23 Fig — For the best by f3 dataset, using the same source populations as in the corresponding MOSAIC analysis. Only populations that Malder estimated had one event are shown. The populations for which Malder inferred two admixture events were: Egypt_Egyptia 40 and 6 generations ago, Sudan_Halfawieen 87 and 7 generations ago, and Sudan_Mahas 94 and 12 generations ago. The blue line is the fitted linear regression line and the grey area represents the 95% confidence interval of the standard error. (PDF) [file pone.0290423.s029.pdf]

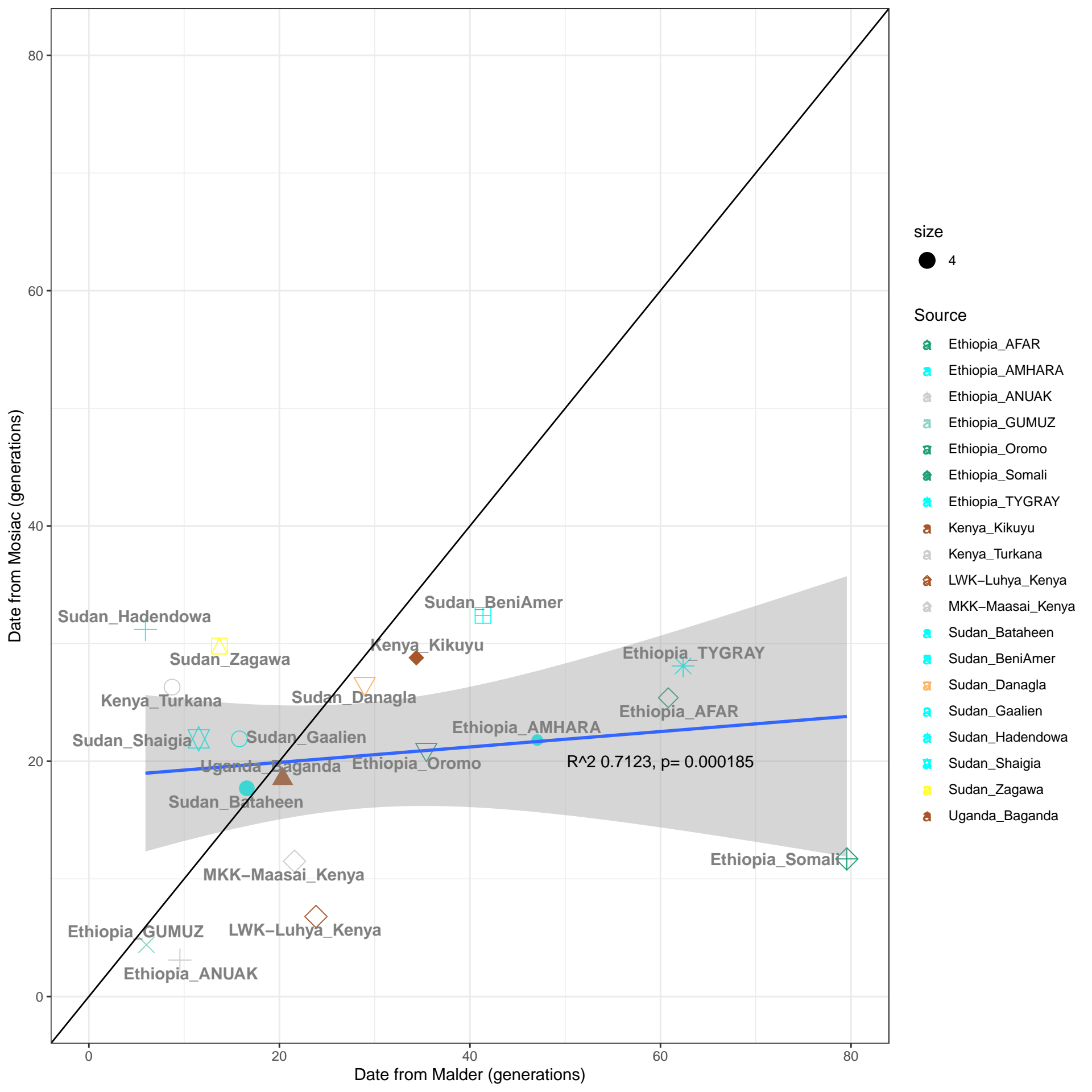

Supplement: S24 Fig — For the best by R2 dataset, using the same source populations as in the corresponding MOSAIC analysis. Only populations that Malder estimated had one event are shown. The populations for which Malder inferred two admixture events were: Egypt_Egyptian 39 and 8 generations ago, Kenya_Turkana 8 and 164 generations ago, Sudan_Halfawieen 77 and 6 generations ago, and Sudan_Mahas 81 and 12 generations ago. The blue line is the fitted linear regression line and the grey area represents the 95% confidence interval of the standard error. (PDF) [file pone.0290423.s030.pdf]
