# Supplementary material for: Eurasian back-migration into Northeast Africa was a complex and multifaceted process
Source: PLoS One. 2023 Nov 8;18(11):e0290423. doi: 10.1371/journal.pone.0290423 (PMC10631636; doi:10.1371/journal.pone.0290423)
Supplement: S4 Fig — Labels are by country and colouring by linguistic family. African_Semitic was used just more easy to distinguish between the investigated populations (target) and the Middle Eastern Semitic populations. Made with Natural Earth. (PDF) [file pone.0290423.s010.pdf]

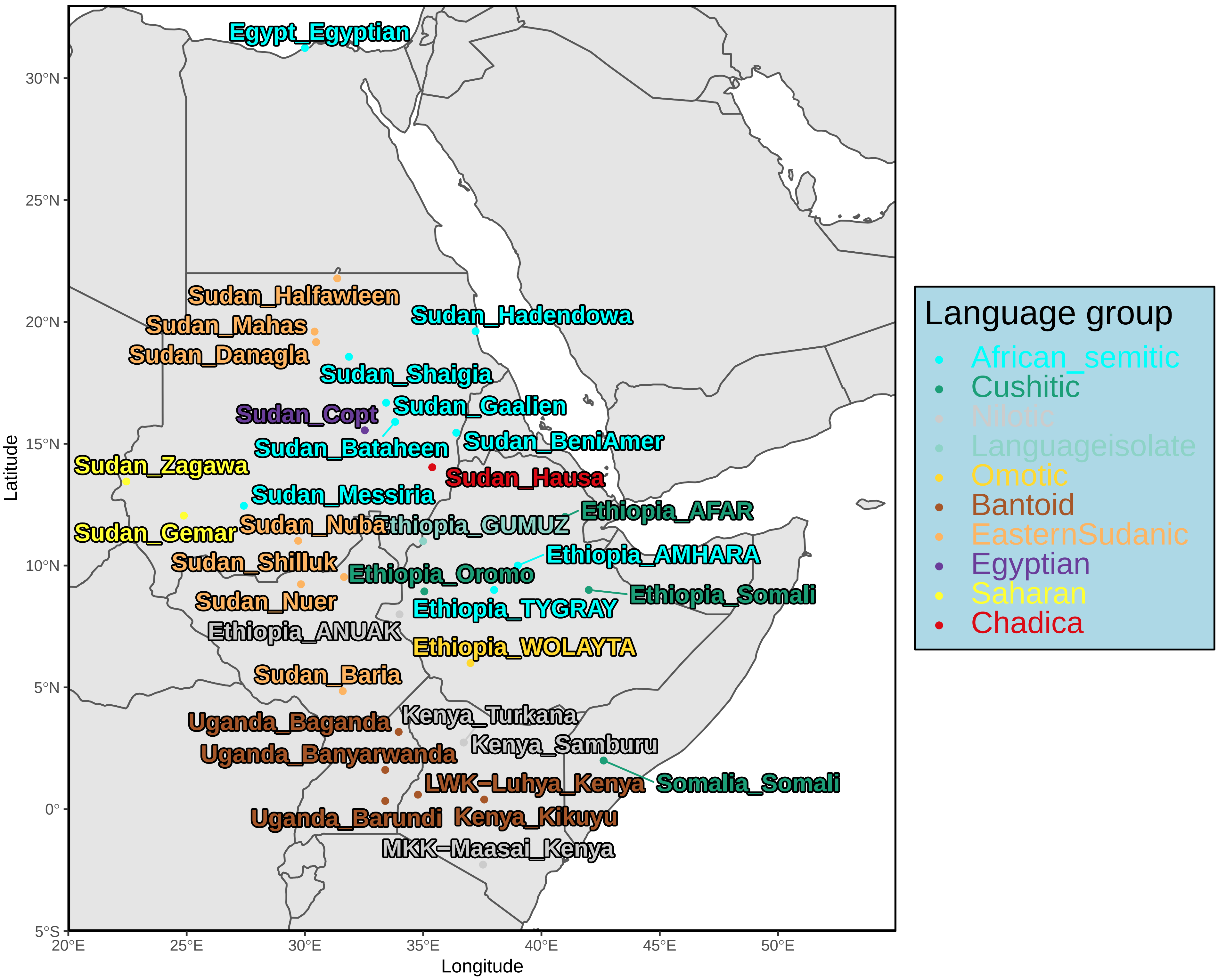
Egypt_Egyptian
Sudan_Halfawieen
Sudan_Hadendowa
Sudan_Mahas
Sudan_Danagla
Sudan_Shaigia
Sudan_Copt
Sudan_Gaalien
Sudan_Bataheen
Sudan_BeniAmer
Sudan_Zagawa
Sudan_Hausa
Sudan_Messiria
Ethiopia_AFAR
Sudan_Gemar
Sudan_Nuba
Ethiopia_GUMUZ
Ethiopia_AMHARA
Sudan_Shilluk
Ethiopia_Oromo
Ethiopia_Somali
Sudan_Nuer
Ethiopia_TYGRAY
Ethiopia_ANUAK
Ethiopia_WOLAYTA
Sudan_Baria
Uganda_Baganda
Kenya_Turkana
Kenya_Samburu
Uganda_Banyarwanda
LWK−Luhya_Kenya
Somalia_Somali
Uganda_Barundi
Kenya_Kikuyu
MKK−Maasai_Kenya
30°N
25°N
20°N
15°N
10°N
5°N
0°
5°S
20°E
25°E
30°E
35°E
40°E
45°E
50°E
Latitude
Longitude
Language group
African_semitic
Cushitic
Nilotic
Languageisolate
Omotic
Bantoid
EasternSudanic
Egyptian
Saharan
Chadica
