# Supplementary material for: Eurasian back-migration into Northeast Africa was a complex and multifaceted process
Source: PLoS One. 2023 Nov 8;18(11):e0290423. doi: 10.1371/journal.pone.0290423 (PMC10631636; doi:10.1371/journal.pone.0290423)
Supplement: S16 Fig — (PDF) [file pone.0290423.s022.pdf]

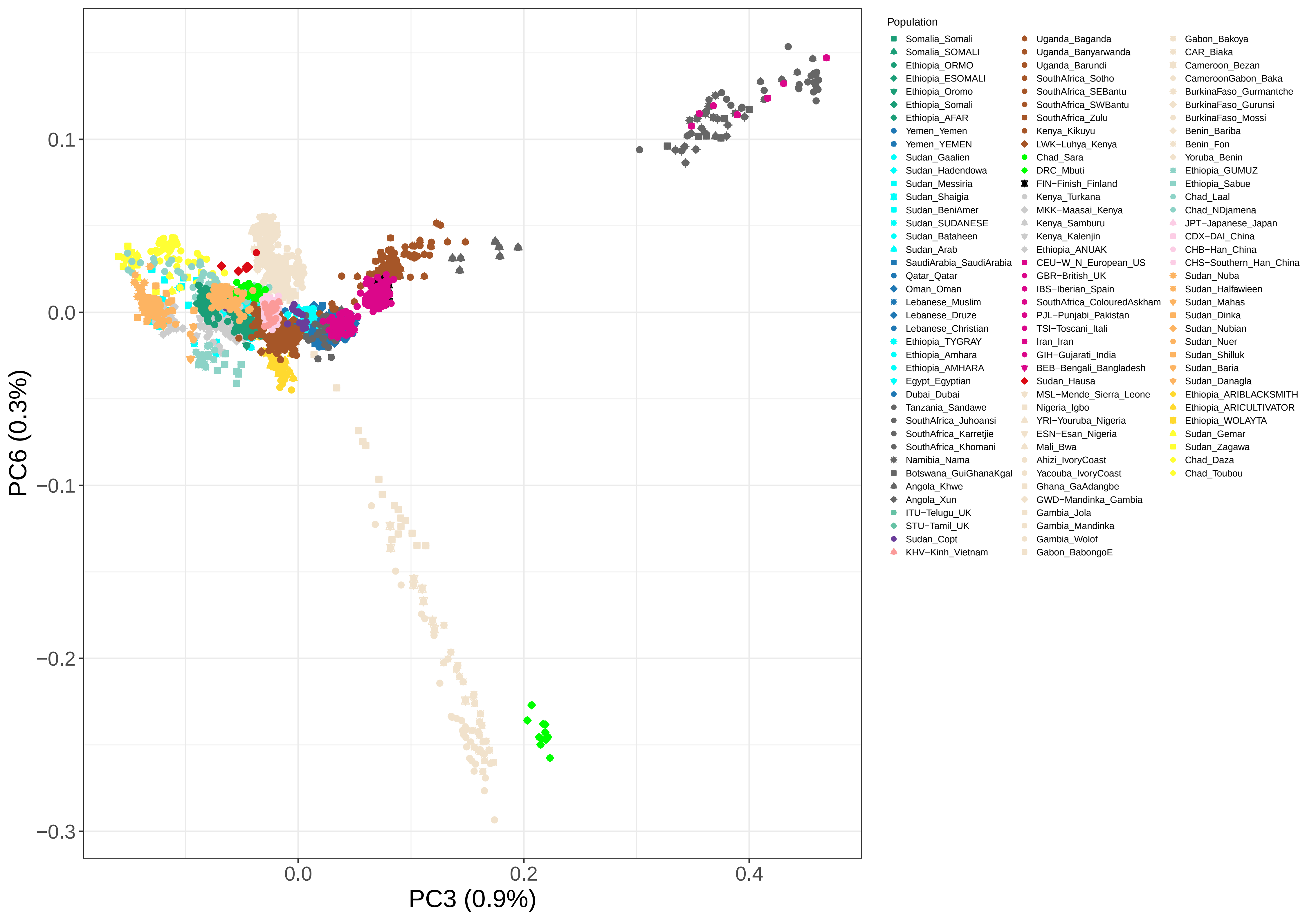

PC6 (0.3%)
PC3 (0.9%)
0.1
0.0
−0.1
−0.2
−0.3
0.0
0.2
0.4
Population
Somalia_Somali
Somalia_SOMALI
Ethiopia_ORMO
Ethiopia_ESOMALI
Ethiopia_Oromo
Ethiopia_Somali
Ethiopia_AFAR
Yemen_Yemen
Yemen_YEMEN
Sudan_Gaalien
Sudan_Hadendowa
Sudan_Messiria
Sudan_Shaigia
Sudan_BeniAmer
Sudan_SUDANESE
Sudan_Bataheen
Sudan_Arab
SaudiArabia_SaudiArabia
Qatar_Qatar
Oman_Oman
Lebanese_Muslim
Lebanese_Druze
Lebanese_Christian
Ethiopia_TYGRAY
Ethiopia_Amhara
Ethiopia_AMHARA
Egypt_Egyptian
Dubai_Dubai
Tanzania_Sandawe
SouthAfrica_Juhoansi
SouthAfrica_Karretjie
SouthAfrica_Khomani
Namibia_Nama
Botswana_GuiGhanaKgal
Angola_Khwe
Angola_Xun
ITU−Telugu_UK
STU−Tamil_UK
Sudan_Copt
KHV−Kinh_Vietnam
Uganda_Baganda
Uganda_Banyarwanda
Uganda_Barundi
SouthAfrica_Sotho
SouthAfrica_SEBantu
SouthAfrica_SWBantu
SouthAfrica_Zulu
Kenya_Kikuyu
LWK−Luhya_Kenya
Chad_Sara
DRC_Mbuti
FIN−Finish_Finland
Kenya_Turkana
MKK−Maasai_Kenya
Kenya_Samburu
Kenya_Kalenjin
Ethiopia_ANUAK
CEU−W_N_European_US
GBR−British_UK
IBS−Iberian_Spain
SouthAfrica_ColouredAskham
PJL−Punjabi_Pakistan
TSI−Toscani_Itali
Iran_Iran
GIH−Gujarati_India
BEB−Bengali_Bangladesh
Sudan_Hausa
MSL−Mende_Sierra_Leone
Nigeria_Igbo
YRI−Youruba_Nigeria
ESN−Esan_Nigeria
Mali_Bwa
Ahizi_IvoryCoast
Yacouba_IvoryCoast
Ghana_GaAdangbe
GWD−Mandinka_Gambia
Gambia_Jola
Gambia_Mandinka
Gambia_Wolof
Gabon_BabongoE
Gabon_Bakoya
CAR_Biaka
Cameroon_Bezan
CameroonGabon_Baka
BurkinaFaso_Gurmantche
BurkinaFaso_Gurunsi
BurkinaFaso_Mossi
Benin_Bariba
Benin_Fon
Yoruba_Benin
Ethiopia_GUMUZ
Ethiopia_Sabue
Chad_Laal
Chad_NDjamena
JPT−Japanese_Japan
CDX−DAI_China
CHB−Han_China
CHS−Southern_Han_China
Sudan_Nuba
Sudan_Halfawieen
Sudan_Mahas
Sudan_Dinka
Sudan_Nubian
Sudan_Nuer
Sudan_Shilluk
Sudan_Baria
Sudan_Danagla
Ethiopia_ARIBLACKSMITH
Ethiopia_ARICULTIVATOR
Ethiopia_WOLAYTA
Sudan_Gemar
Sudan_Zagawa
Chad_Daza
Chad_Toubou
