# Supplementary material for: Eurasian back-migration into Northeast Africa was a complex and multifaceted process
Source: PLoS One. 2023 Nov 8;18(11):e0290423. doi: 10.1371/journal.pone.0290423 (PMC10631636; doi:10.1371/journal.pone.0290423)
Supplement: Attachment — Submitted filename: NEA revisions 28 Febr.pdf [file pone.0290423.s031.pdf]

Dear Editor

Thank you for the constructive and encouraging set of reviews of our manuscript "*Eurasian back-migration into Northeast Africa was a complex and multifaceted process*". The constructive feedback that the referees provided was very helpful. We now updated the manuscript and incorporated the suggested improvements. In the letter below, we include responses to each of the referees' comments and describe how the paper has changed as a result. The original comments of the reviewers are pasted below and our reply follows the "Author reply:" statement. We also updated the manuscript following the editors' recommendations regarding the format. We also would like to note that we do indeed make use of the Natural Earth public domain material. We specified this now clearly below the figure where it is implemented.

Reviewer comments:

Reviewer 1:

Reviewer #1: The manuscript by Hammaren and colleagues addresses an interesting question about North African populations: the origin of the Eurasian back-migrations. I highly appreciate that the authors reanalyzed data from previous papers that could be further explored. However, the answer to the main question is not clearly discussed. The paper would improve with a clearer discussion.

Author reply: We would like to thank the reviewer for their positive evaluation of our manuscript. We re-wrote and re-structured large parts of the discussion and hope that the readability increased.

1. In general, there is too much emphasis and sometimes overinterpretation of the ADMIXTURE analyses. The discussion in mainly driven by those results, but the most novel and interesting part are the f3 analyses after local ancestry:

   - The text is too descriptive and difficult to follow. Some of the descriptions about ADMIXTURE components and PC could be shortened.

Author reply: Thank you for pointing this out - we agree with the reviewer and the results and discussion section describing population structure was shortened and streamlined.

2. You might need to review the colors linked to the ADMIXTURE plots, sometimes they do not match with the description in the text:

-"Light green component that emerges is K=7 it is maximized in the Sabue". This color is maximed in Namibia_Nama SouthAfrica_Juhoansi.

-"blush pink component highest in the Qatari". It is higher in Yemen.

-“Gumuz and Sabue have a high proportion of the dark green component.” Is this the black component?

-“blue component maximized in Middle Eastern groups at K=7, Figure 2.” I think this is figure 1.

Author reply: This section was re-written in shorter format and the colors were corrected

3- Also, you might want to check the labels of the ADMIXTURE plots, the names are sometimes duplicated and not clear.

Author reply: We updated ADMIXTURE plots in both Figure 1 and figure S2. We improved the label text of both

4- I would suggest providing a visualization of the f3 results in Table 1. And discussing these results and their interpretations in the discussion.

Author reply: Thank you for the recommendation. We now did a visualization of the f3 results added as main figure 2 and also discussed this figure in the text. We appreciate the reviewers’ suggestion regarding this since some patterns became more apparent after visualization.

5- Regarding ADMIXTURE interpretations, one example is the hunter-gatherer ancestry, which is mentioned through the text at different points. Are you claiming that it corresponds to one of their ancestral components in ADMIXTURE? It is too speculative without ancient data. Also, talking about “ancestries” from ADMIXTURE analyses is in most cases speculative, I would suggest checking these interpretations and the language used.

Author reply: We now have re-written these sections and clarified the language. We also clarified the connection of East African hunter-gatherer ancestries to the ancient DNA Mota genome, which was made by previous aDNA studies that we include as citations in the text.

6- In the discussion, the paragraph “Previous recent ancestry deconvolution studies pointed at Levantine”. You are repeating the description results that were already mentioned, but it is not clear what is the interpretation.

Author reply: This was explained further. The patterns are also now clearer with f3 visualizations in Figure 2

About the methodology:

7- It is not explained how you treat the data after the local ancestry. Do you mask the rest of the genomes setting it as missing? If that is the case, which proportion of the genome is masked and therefore what are the missingness values?

Author reply: The missingness values were added in Table S5. It is now also clarified in the text that the rest of the genome is set to missing.

8- Could the use of CEU for the local ancestry bias the following analyses about the specific source for this ancestry? It is good that the analyses are repeated with the best source from f3 afterwards, but I wonder if this could produce a circular result (i.e. if the first analyses using CEU are biasing the f3 estimates, so the next analyses would be also biased). Could you comment it further?

Author reply: We agree that circularity might be a pitfall - we therefore try to avoid circularity by excluding CEU from the second cycle. The best-by-R2 approach is also an attempt to circumvent this.

9- You mention: “Though using two Eurasian populations as sources outperformed a single source.” Is it possible that this happens because you do not have the best source and then it is more likely that the source is coming from two different ones?

Author reply: We agree with the reviewer. We added a sentence “This could be because our dataset does not contain a good match to the original source”

10- Is it possible to estimate confidence intervals for the admixture dating? Considering the differences observed between the “f3” and “R2” estimations it would be important. The correlation between both admixture dates is not strongly significant and there are some outliers. It is briefly disused in the discussion, but why specifically do you think these differences are found? For instance, is there the worst correlation among those with more Eurasian ancestry?

Author reply: For both datasets we investigated this aspect further by plotting the residuals to the fitted line vs. Eurasian fraction. We found contrasting results for the two datasets and we include them as reviewer files. We however did not add them to the manuscript as we thought these comparisons will not add significant discussion points to the manuscript.

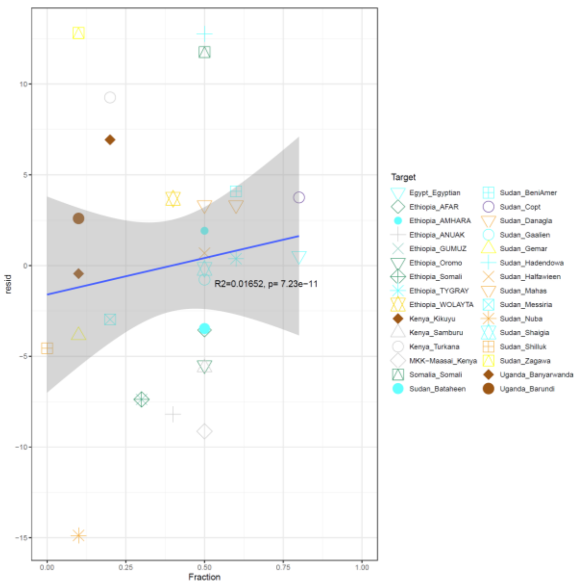

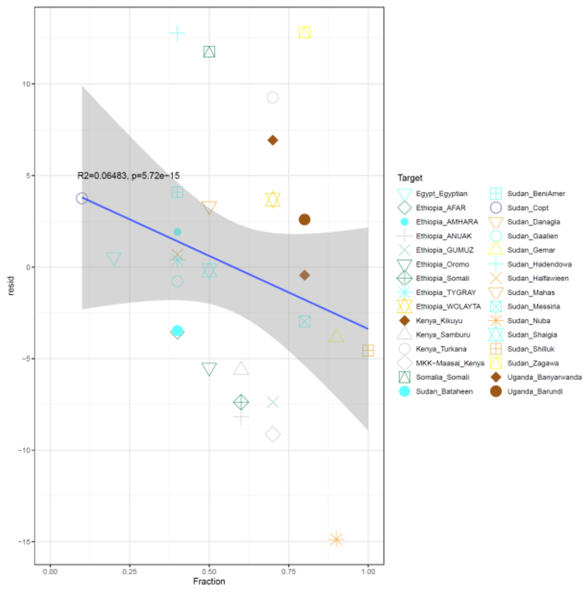

11- To see if the masking is necessary, I suggest using an f4 test comparing between possible sources of the Eurasian ancestry in the different Northeast African populations, i.e. f4(Levant, Arab; Northeast Africa, Ju'hoansi).

Author reply: We added f4 estimations in figure S22 and added a paragraph in the text.

12- It would be interesting to see the ancestry proportions estimated with MOSAIC. For instance, comparing them with the admixture dates could bring more information about the different demographic processes.

Author reply: We compared the ancestry proportion of Eurasian-like ancestry and the inferred dates of admixture for the two datasets respectively. There was a weakly significant positive correlation with low R2 values for the best by f3 dataset. For the best by R2 dataset there was a significant negative correlation. We attach these two comparisons to the re-submission files. We however did not add them to the manuscript as they gave contradicting results for the two methods and we thought these comparisons will not add significant discussion points to the manuscript.

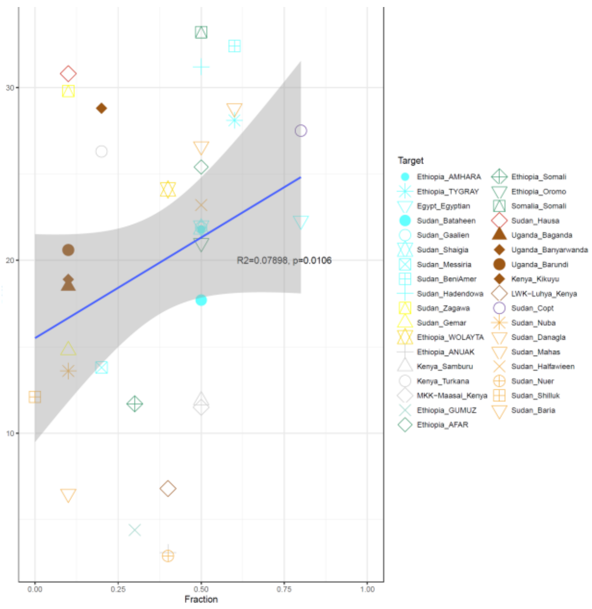

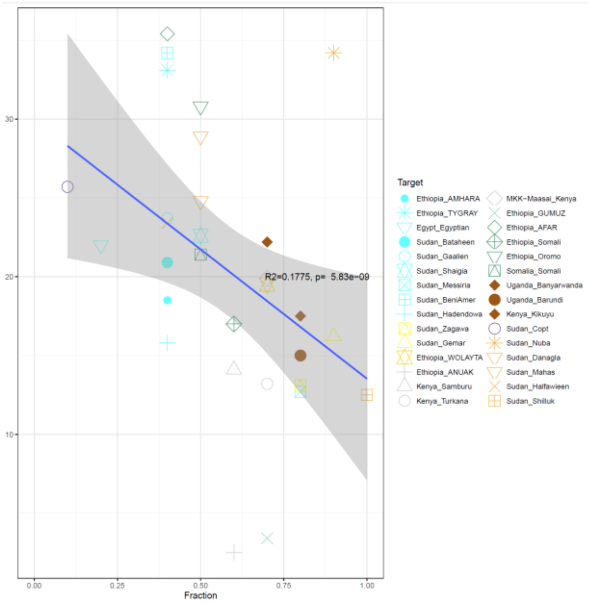

13- What are the sources of the admixture dating, is it always the Eurasian population and an African population? For instance, in the discussion, when talking about the Copts and Egyptians, it is not clear what are the sources of the admixture dates described.

Author reply: Yes it is always the Eurasian admixture that we consider. This was indicated in the first sentence of the “Dating Eurasian admixture in Northeast Africa” section in the Results section:

“For both the f3 outgroup-based approach and the R2 approach above, we determined the admixture date (in generations) from MOSAIC’s co-ancestry curves for the Eurasian-like constructed ancestry”

We also now clarified this in the section where the admixture into the Copts was discussed.

14- The discussion about how more recent admixture events could be masking the older ones is not supported with specific analyses. Maybe the authors could cite at least some other works.

Author reply: We now cite the ancient DNA study of Prendergast et al in the Conclusions section, which inferred older admixture dates based on aDNA data.

15- There are some other places where a reference would help, for instance, “The pattern is true also for the rest of North Africa, though not investigated here.”

Author reply: The reference was in the next sentence and the sentence is now merged for clarity sake

16-On a minor note, English is readable, but needs to be checked. There are some repetitions of words and gramma mistakes. I am not listing them because there are many, so please check it carefully. Some that I found:

Author reply: We did a thorough check of the manuscript to correct grammar mistakes

17-"are evident across the region of interest interest we used the FEEMS"

Author reply: We fixed this and also did a thorough check of the rest of the manuscript to correct grammar mistakes

18-The title in the results: "Dating Eurasian admixture dating in Northeast Africa." You do not need the second "dating".

Author reply: Fixed- thank you

Reviewer 2:

In the manuscript by Hammaren et al the authors attempt to disentangle some of the complexity in the genetic history of present day populations from North East Africa. They begin by collecting a large dataset of publicly available present-day data spanning all of Africa, the Near East and Europe, and then apply a variety of methods in attempts to identify present-day populations that appear distinctively similar to the introgressed Eurasian fragments in North East African groups and could thus be good proxies, or at least shed light onto the past dynamics of the region.

Although their final conclusion is that their attempt is not as successful as they would like, it does take time to get there, and I think this is something that could be made clearer earlier on, although I also grant it might be a different in writing style. But personally, I found the concluding remarks somewhat undermined the rest of the discussion, for all that I very much agree with the key points made in that final section, and I wonder if it might be more effective if brought in earlier. Beyond that, I have mostly minor comments, and no concerns to raise about the scientific content of the manuscript as much as about its presentation.

Author reply: We thank the reviewer for their in-depth review of our manuscript. We restructure the discussion and we hope it improved the readability

1. Title: should be either "Eurasian back-migrations into Northeast Africa WERE a complex and multifaceted process" or "Eurasian back-migration into Northeast Africa was a complex and multifaceted process"; the current version doesn't have subject/tense agreement.

Author reply: Fixed - thank you

2. Methods, "4) individuals with at least 10% missingness was removed (plink --mind 0.01)" That is actually a filter for 1% missingness - please confirm what was actually done!
Author reply: Fixed - thank you. We re-checked and it was actually –mind 0.15 so 15%

3. Results, "Note that some populations are represented multiple times from different original publications, resulting in a total of 97 unique populations." As a sanity check for the dataset merging, do results for the same population across different publications generally agree?
Author reply: We checked this and they agree very well in all the population structure analyses. For the main analyses however, we only used one of each.

4. Results, "The output from Admixture shown in Figure 1 (for full analysis see Supplementary Figure 2)" It would be good if the figure legend (or somewhere in the main text) made clear that this is a truncated version of Supplementary Figure 2, and the K=13 and 14 panels don't include all clusters (East Asia is missing), as the 'full analysis' is a bit ambiguous here, since not all values of K are shown in the main text fig. Likewise, an explanation of what 'w' is in panel 1D would be helpful for readers not very familiar with FEEMS.
Author reply: The legend was updated and it now explains that 1A is a truncated version. It also clarifies the "w" symbol in FEEMS analyses.

5. Results, "Ancestry tract length distribution plots for both of these datasets was generated and are available on request." If this is a matter of just plots, please add them as supplements to begin with. It'll be easier for everyone than having to dig them up when a reader asks for them in three years!
Author reply: Since there are 70 figures we chose to not include this in the supplementary data

6. Discussion, "Admixture analyses recapitulate this pattern where Northeast African groups share the blue component maximized in Middle Eastern groups at K=7, Figure 2." That should surely be figure 1?
Author reply: Fixed - thank you - it should have been Figure S2

7. Discussion, "Dongola had been the capital of the Nubian Kingdom and the fall of Dongola in 317 to Mameluke forces meant the start of Arab and Islamic dominance south of the borders of Egypt. Many of the Semitic speakers in our dataset have their Eurasian admixture dated to this time - around 20+ generations ago.", Is there a typo here, or a number missing? Using the value given in the text of 29 years per generation, 20 generations is 580 years ago, and looking at the corresponding figure no numbers are anywhere near close to the 58 generations needed to get to get to 320.
Author reply: Fixed - correct date is 1317, a "1" was omitted

8. Discussion, "One possible explanation for this phenomenon could be that populations with little or no previous Eurasian admixture would have their inferred admixture date effected more by recent

Eurasian admixture than population that experienced larger admixture in the past." Effected should be affected.
Author reply: Fixed - thank you

Reviewer 3:

In “Eurasian back-migrations into Northeast Africa was a complex and multifaceted process” Rickard Hammaren and colleagues assembled and analysed a genome-wide dataset composed of XXX individuals from several populations focusing on the recent demographic dynamics of the North-Eastern side of the African continent.

In doing so, they apply different population structure and local ancestry analyses. The main claim the authors make is that “the distribution of the Eurasian-like ancestry in the Eastern an North-Eastern African populations is mostly an effect of more recent migrations rather than ancient events related to the advent of pastoralism in the region at large”.

1. In my opinion this is an interesting but at the same time controversial statement, which requires a carfeul validation analysis. A series of different local ancestry analysis and admixture dating have repeatedly reported a signal of Eurasian-like ancestry entering into the continent at least 3,000 years ago. Although I appreciate the fact that the complexity of the many layers intersecting since at least 15,00 years ago may introduce many bias in previous researches, I can’t see how the analysis conducted in this paper could not affected by similar issues. Moreover, I have previously attempted to use MOSAIC on simulated data, and in our specific setting, MOSAIC was not able to infer the true ancestral fragments, causing a bias in the admixture dating. I did not conduct any further analysis and therefore I do not have any evidence MOSAIC does something wrong, but I would definitely use some other local ancestry inference methods and admixture dating approaches checking if the results hold.

Author reply: We thank the reviewer for their thorough assessment of the manuscript. We compared our admixture dating inferences with inferences using the MALDER software. We added these as figures S23 and S24. We found a much stronger and significant correlation between our Best by R2 approach and MALDER dates. We comment on this in the text.

Minor considerations:

2.Pag: I would clearly state the number of individuals and populations in the final dataset
Author reply: We indicated this in the first part of the Results section:
“After quality control, removal of related individuals, and down-sampling to a maximum of 30 individuals per population the dataset consisted of 2066 individuals from 101 population groups and 199 422 SNPs.”

Pag 3: it is not clear if you removed individuals with missing exceeding 1% or 10% as the description and the command line are discordant.

Author reply: Fixed - thank you. We re-checked and it was actually –mind 0.x15 so 15%

Pag 3: I think that the UMAP analysis should be described in a more detailed way, and possibly evaluating the outcome of multiple iterations.
Author reply: We ran several iterations and they gave very similar outcomes. The rotation is different each time but the overall shape was very similar.

Pag 4: Maybe F3 could be written as f3 ?
Author reply: We changed F3 to f3 throughout the text

Pag 5: Given that this article might be of interest to many scholars, I would specify what TSI and RHGs mean.
Author reply: This is now fixed in the text - thank you

Pag 6: The last paragraph explaining the approach for the best R2 and f3 was, in my opinion hard to read, I would consider reformulating it.
Author reply: We updated the text and tried to clarify the procedure better

Pag 7: I think that the Discussions are too long, containing many paragraphs that would probably be a better fit into the results section.
Author reply: We reordered the discussion and moved some paragraphs to the Results section
